# β-Glutamine-mediated self-association of transmembrane β-peptides within lipid bilayers†
†Electronic supplementary information (ESI) available: Material and methods, syntheses and characterizations of the β^3^-amino acids and the β-peptides, enantiomeric purity, CD and UV spectroscopic methods, fluorescence emission spectra. See DOI: 10.1039/c6sc01147k


**DOI:** 10.1039/c6sc01147k

**Published:** 2016-05-19

**Authors:** U. Rost, C. Steinem, U. Diederichsen

**Affiliations:** a Institute of Organic and Biomolecular Chemistry , Georg-August-University Goettingen , Tammannstr. 2 , 37077 Goettingen , Germany . Email: csteine@gwdg.de ; Email: udieder@gwdg.de

## Abstract

Transmembrane β-peptide helices and their association in lipid membranes are still widely unexplored. We designed and synthesized transmembrane β-peptides harboring different numbers of d-β^3^-glutamine residues (^h^Gln) by solid phase peptide synthesis. By means of circular dichroism spectroscopic measurements, the secondary structure of the β-peptides reconstituted into unilamellar vesicles was determined to be similar to a right-handed 3_14_-helix. Fluorescence spectroscopy using d-β^3^-tryptophan residues strongly suggested a transmembrane orientation. Two or three ^h^Gln served as recognition units between the helices to allow helix–helix assembly driven by hydrogen bond formation. The association state of the transmembrane β-peptides as a function of the number of ^h^Gln residues was investigated by fluorescence resonance energy transfer (FRET). Therefore, two fluorescence probes (NBD, TAMRA) were covalently attached to the side chains of the transmembrane β-peptide helices. The results clearly demonstrate that only β-peptides with ^h^Gln as recognition units assemble into oligomers, presumably trimers. Temperature dependent FRET experiments further show that the strength of the helix–helix association is a function of the number of ^h^Gln residues in the helix.

## Abstract 2

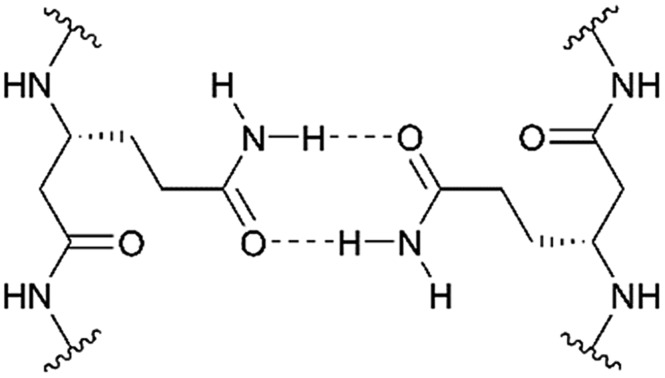
The rational design and synthesis of novel transmembrane β-peptides forming stable secondary structures in a membrane environment are described. Their state of aggregation within the membrane is controlled by hydrogen bonds.

## Introduction

In the last three decades, there has been great interest in the design and synthesis of unnatural β-peptide oligomers in solution, which are capable of forming stable secondary structures and which are resistant to proteolytic degradation by proteases and peptidases.^1^,^2^ Folding into well-defined secondary structures is a fundamental part of protein architecture and takes part in the recognition process between peptides.^3^ Even though integral membrane proteins represent nearly 30% of the human proteome and are involved in various cellular processes, the complexity of these protein structures often limits the precise analysis of their biological activities, functions and mechanisms on the molecular level.^4^–^6^ To accomplish their function, membrane proteins can either be monomeric or they need to be assembled into oligomeric structures. Studies on the fundamental molecular aspects of membrane protein oligomerization have been mainly performed using model systems of transmembrane α-peptide helices.^5^–^9^ The organization and assembly of these transmembrane α-peptide helices depend on the lipid environment and the interacting molecular species themselves. These interactions are still not fully understood and synthetically readily available transmembrane peptide models are an appreciated tool to investigate peptide–lipid and peptide–peptide interactions occurring in membrane associated processes.^6^ However, in contrast to membrane embedded α-peptide helices the field of transmembrane β-peptide helices and their association within a membrane is still widely unexplored. In contrast to α-peptides, β-peptides have one additional CH_2_-group inserted in every amino acid residue, which offers an increased number of possible conformers. β-Peptides are known to form stable secondary structure elements even with short chain lengths (>6 amino acid residues) and they allow the width of the helix to be varied, as well as the helical dipole moment.^10^ Appella *et al.* demonstrated that small 10-residue amphiphilic β-peptides form water soluble aggregates.^11^,^12^ In addition, specific β-peptide helix interactions in solution, controlled by the attachment of nucleobase pairing recognition units, have been reported.^3^,^4^

**Fig. 1:**
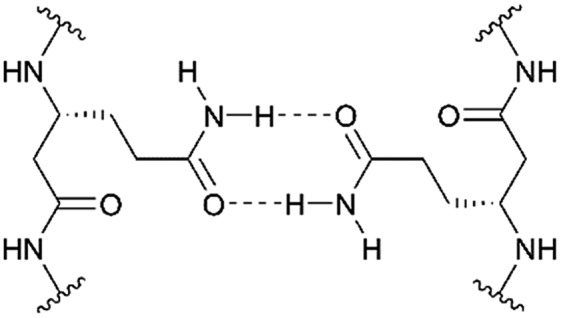
Schematic illustration of hydrogen bond formation of d-β^3^-glutamine residues.

In the present study, we describe the rational design and synthesis of novel transmembrane β-peptides forming stable secondary structures in a membrane environment as analysed by means of circular dichroism (CD) spectroscopy. Membrane insertion was verified by fluorescence spectroscopy detecting the intrinsic tryptophan fluorescence.^13^,^14^ The transmembrane β-peptide helices were designed to allow for peptide oligomerization based on hydrogen bond formation within a lipid bilayer. Hydrogen bond formation is established by placing d-β^3^-glutamine (Fig. 1) at defined positions within the β-peptide in analogy to asparagine, which has been shown to form hydrogen bonds and thus allows for a self-association of α-peptide helices.^9^,^15^ The peptide–peptide association of the synthesized β-peptides reconstituted into lipid bilayers was investigated by fluorescence resonance energy transfer (FRET), which is a powerful and widely used technique to analyse association processes.^16^,^17^

## Results and discussion

### Design of the transmembrane β-peptides

**Fig. 2:**
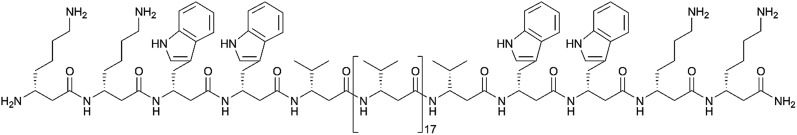
Basic structure of the designed β-peptides (H-^h^Lys_2_-^h^Trp_2_-^h^Val_19_-^h^Trp_2_-^h^Lys_2_-NH_2_).

**Fig. 3:**
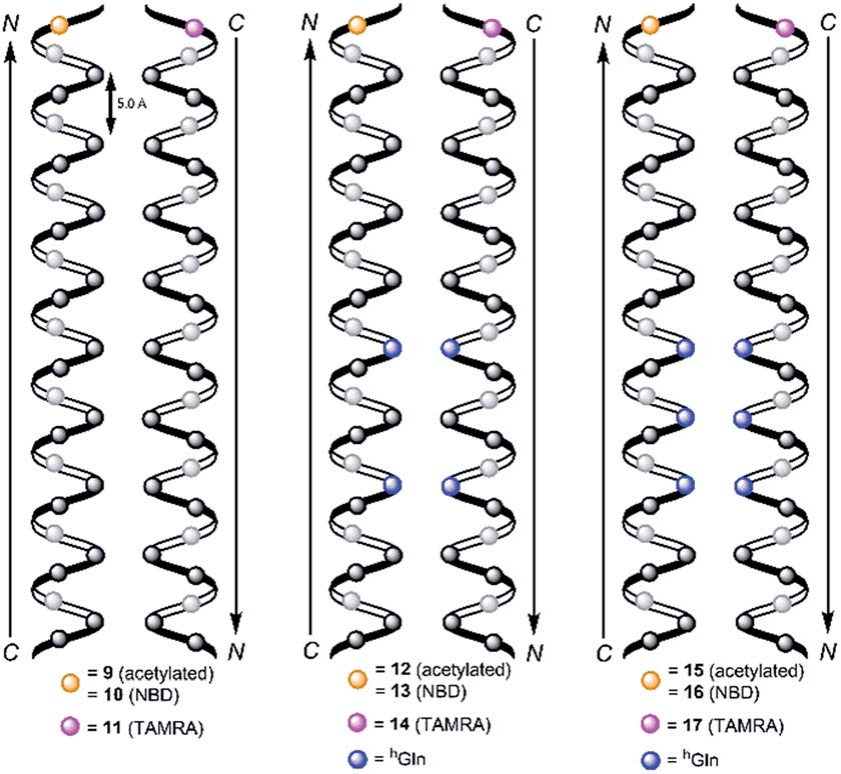
Schematic view on two 3_14_-helical β-peptides oriented in an antiparallel fashion with three amino acids forming one turn and zero ^h^Gln (**9**/**10**/**11**, left), two ^h^Gln (**12**/**13**/**14**, middle) and three ^h^Gln (**15**/**16**/**17**, right) recognition units. The β-peptides were acetylated or labelled with NBD and TAMRA for FRET-analysis and synthesized from N- to C-terminus.

We followed the approach to synthesize β-peptides that are expected to form a 3_14_-helix and can be inserted into lipid bilayers in a transmembrane configuration. In a 3_14_-helix, three amino acid residues form one helix turn in which every fourth amino acid side chain is oriented on the same face of the helix.^4^,^18^ To span the hydrophobic core of a lipid bilayer, the β-peptides were composed of 19 d-β^3^-valines.^19^–^22^ Considering the peptide–lipid interactions, the lipid–water interface plays a vital role. Therefore, the β-peptides were flanked with two d-β^3^-tryptophan residues at the N- and C-terminal end of the sequence (Fig. 2). The aromatic indole moieties of these d-β^3^-tryptophans are known to arrange in the polar/apolar interface of the lipid membrane.^23^ While the aromatic ring is preferentially localized in the apolar part of the membrane, the indole NH groups are adjacent to the lipid carbonyl moiety, where they form hydrogen bonds *via* the NH-group to anchor and stabilize the peptide in the lipid membrane.^24^–^26^ Furthermore, two d-β^3^-lysine residues were attached at each side of the β-peptides to increase their solubility. It is known that lysine residues with long and flexible side chains facilitate the solubility in aqueous systems.^23^ In total, we designed the β-peptide H-^h^Lys_2_-^h^Trp_2_-^h^Val_19_-^h^Trp_2_-^h^Lys_2_-NH_2_ as a core unit for our studies (Fig. 2). d-β^3^-Glutamine was introduced as a recognition unit to control transmembrane β-peptide helix aggregation *via* hydrogen bond formation. We have chosen d-β^3^-glutamine, as its side chain has one additional CH_2_-group, which increases the side chains' flexibility compared to asparagine. d-β^3^-Valine residues were replaced by two or three d-β^3^-glutamines. Nucleobase functionalized β-peptides are known to prefer an antiparallel strand orientation in aqueous solutions.^3^,^4^ Hence, we anticipate that the transmembrane β-peptides also associate in an antiparallel fashion and placed the respective d-β^3^-glutamine residues accordingly (Table 1, Fig. 3). In order to perform FRET-analysis, two different fluorophores (NBD, TAMRA), serving as a donor–acceptor pair, were attached to the β-peptides. As free peptide termini are required for membrane insertion, and in order to avoid any conformational restrictions as a result of fluorophore labelling, the fluorophores were attached to the side chain of an additional d-β^3^-lysine residue that was added either to the C-terminal or the N-terminal end depending on the β-peptide sequence.^6^

**Table 1:** Synthesized transmembrane β-peptides with zero, two or three d-β^3^-glutamine recognition units

No.	Synthesized transmembrane β-peptides
**Zero recognition units**	
**9**	H-^h^Lys(acetyl)-^h^Lys_2_-^h^Trp_2_-^h^Val_19_-^h^Trp_2_-^h^Lys_2_-NH_2_
**10**	H-^h^Lys(NBD)-^h^Lys_2_-^h^Trp_2_-^h^Val_19_-^h^Trp_2_-^h^Lys_2_-NH_2_
**11**	H-^h^Lys_2_-^h^Trp_2_-^h^Val_19_-^h^Trp_2_-^h^Lys_2_-^h^Lys(TAMRA)-NH_2_

**Two** ^**h**^ **Gln recognition units**	
**12**	H-^h^Lys(acetyl)-^h^Lys_2_-^h^Trp_2_-^h^Val_9_-^h^Gln-^h^Val_5_-^h^Gln-^h^Val_3_-^h^Trp_2_-^h^Lys_2_-NH_2_
**13**	H-^h^Lys(NBD)-^h^Lys_2_-^h^Trp_2_-^h^Val_9_-^h^Gln-^h^Val_5_-^h^Gln-^h^Val_3_-^h^Trp_2_-^h^Lys_2_-NH_2_
**14**	H-^h^Lys_2_-^h^Trp_2_-^h^Val_3_-^h^Gln-^h^Val_5_-^h^Gln-^h^Val_9_-^h^Trp_2_-^h^Lys_2_-^h^Lys(TAMRA)-NH_2_

**Three** ^**h**^ **Gln recognition units**	
**15**	H-^h^Lys(acetyl)-^h^Lys_2_-^h^Trp_2_-^h^Val_9_-^h^Gln-^h^Val_2_-^h^Gln-^h^Val_2_-^h^Gln-^h^Val_3_-^h^Trp_2_-^h^Lys_2_-NH_2_
**16**	H-^h^Lys(NBD)-^h^Lys_2_-^h^Trp_2_-^h^Val_9_-^h^Gln-^h^Val_2_-^h^Gln-^h^Val_2_-^h^Gln-^h^Val_3_-^h^Trp_2_-^h^Lys_2_-NH_2_
**17**	H-^h^Lys_2_-^h^Trp_2_-^h^Val_3_-^h^Gln-^h^Val_2_-^h^Gln-^h^Val_2_-^h^Gln-^h^Val_9_-^h^Trp_2_-^h^Lys_2_-^h^Lys(TAMRA)-NH_2_

Consequently, three different types of transmembrane β-peptides were designed for analysing β-peptide helix association: (i) **9**/**10**/**11** with zero ^h^Gln, (ii) **12**/**13**/**14** with two ^h^Gln as recognition units and (iii) **15**/**16**/**17** three ^h^Gln as recognition units (Table 1).

### CD-spectroscopic measurements

Structural studies on β-peptides using NMR- and circular dichroism (CD)-spectroscopy, X-ray crystallography as well as molecular dynamic simulation studies have shown that they adopt stable secondary structures in solution.^1^,^27^–^32^ In particular, β-peptides rich in β^3^-valine are well-known to form the 3_14_-helical structure.^21^,^22^ Here, we used CD-spectroscopy to assess the secondary structure of the three synthesized β-peptides.^29^,^33^ First, CD-spectra of the β-peptides **9**, **12** and **15** were examined in 2,2,2-trifluoroethanol (TFE), which is known to stabilize secondary structure formation (peptide concentration 38 μm, 25 °C).^34^,^35^ All three β-peptides show similar and very characteristic CD-spectra with a minimum at 193 nm, a zero crossing at 201 nm and a maximum at 211 nm (ESI Fig. S2†). These bands are indicative of a 3_14_-helix as reported for peptides in solutions.^22^,^28^,^36^ Seebach and co-workers were able to unambiguously demonstrate by a combination of CD-spectroscopy, NMR-spectroscopy, X-ray crystallography and molecular dynamic simulation studies that the characteristic CD bands are a result of a left-handed 3_14_-helical structure in solution.^1^,^29^–^32^ As the recorded CD-spectra of the β-peptides **9**, **12** and **15** are mirrored compared to the published ones by Seebach and co-workers, we conclude that the β-peptides **9**, **12** and **15** fold into right-handed 3_14_-helices. The 3_14_-helix is the most frequently documented secondary structure of folded β-peptides. It consists of 14-membered hydrogen-bonded rings between N–H (*i*) and C=O (*i* + 2) with a three-residue repeating arrangement.^37^

**Fig. 4:**
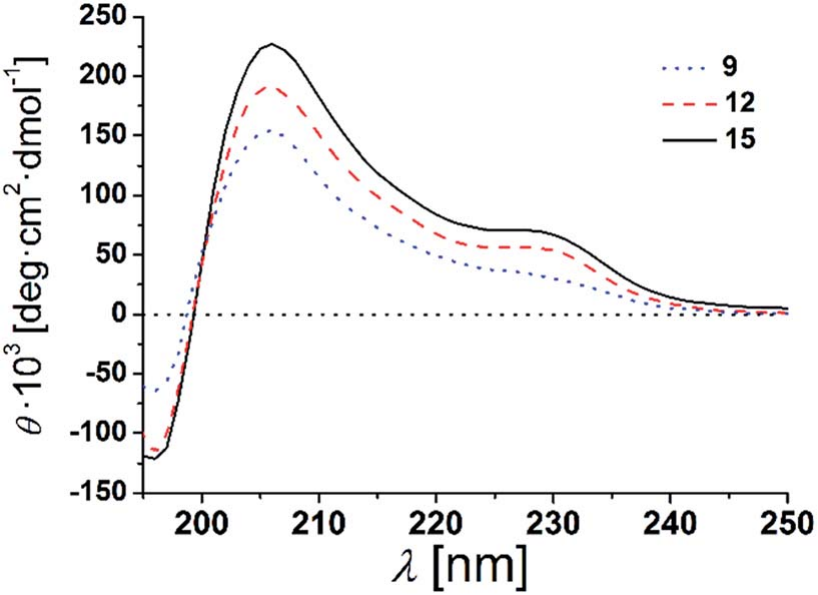
CD-spectra of **9**, **12** and **15** in DOPC LUVs (peptides concentration: 38 μm, P/L-ratio = 1/20, 25 °C).

The β-peptides **9**, **12** and **15** were then reconstituted in a membrane environment, *i.e.* in large unilamellar vesicles (LUVs) composed of DOPC at a β-peptide concentration of 38 μm and a P/L-ratio of 1/20 at 25 °C. Again CD-spectra were recorded to determine the secondary structures (Fig. 4).

The CD-spectra of all three β-peptides show again very similar bands with a minimum at 196 nm, a zero crossing near 199 nm and a maximum at 206 nm indicative of a right-handed 3_14_-helix. Hamuro *et al.* have shown that the presence of micelles strongly stabilizes the 3_14_-helical conformation of amphiphilic β-peptides and the mean residue ellipticity increases in a length-dependent manner consistent with our results.^36^,^38^ Even though the overall signatures of the CD-spectra confirms the right-handed 3_14_-helix, the maxima and minima are slightly shifted compared to the spectra obtained in TFE. The slight shifts of the minima and maxima are a result of the differences in the dielectric constants of the two different solvents, TFE and DOPC lipid (TRIS® buffer).^39^ These findings strongly support our notion that the three β-peptides form a stable secondary structure in the membrane environment. CD-spectroscopy does not only depend on the backbone but also on the side chain conformation.^40^ In helical peptides, aromatic side chains like tryptophan, show characteristic CD values in the 225 nm-region, even if they are positioned near the end of the helix.^41^–^43^ Indeed, in addition to the similar characteristic pattern of the right-handed 3_14_-helix, the presented CD-spectra of the three β-peptides show a weak maximum at 229 nm due to the four d-β^3^-tryptophans, which were attached to the β-peptides (two at each side).

It is expected that the secondary structure of the β-peptides will be very stable, in particular, if embedded in a lipid bilayer. To analyse the thermal stability of the secondary structure, we measured CD-spectra of the β-peptides **12** and **15** reconstituted in DOPC-LUVs (peptide concentration: 38 μm and P/L-ratio = 1/20) at 25 °C and at 60 °C (ESI Fig. S3†). The CD-spectra clearly indicate that the secondary structure of the β-peptides is almost unaffected by the increase in temperature from 25 °C to 60 °C, supporting the thermal stability of the β-peptide secondary structures. Beside the neat secondary structure determination and the thermal stability, the CD-spectra also demonstrate that neither the recognition units nor the acetylated side chain influence the secondary structure of the β-peptides. However, structural conclusions for membrane incorporated β-peptides have only qualitative character since they are mainly based on CD-spectroscopic measurements and comparison with solution structures of 3_14_-helices.

### Tryptophan fluorescence spectroscopy

The two tryptophan residues were placed at both, the N- and C-terminal part of the β-peptides to position them in a transmembrane orientation with the tryptophan residues residing in the polar/apolar interface of the lipid membrane. As Trp-fluorescence is known to be sensitive to the polarity of its local environment, we monitored its maximum *λ*_max_ to determine the β-peptides positions in a lipid bilayer.^13^,^44^

**Fig. 5:**
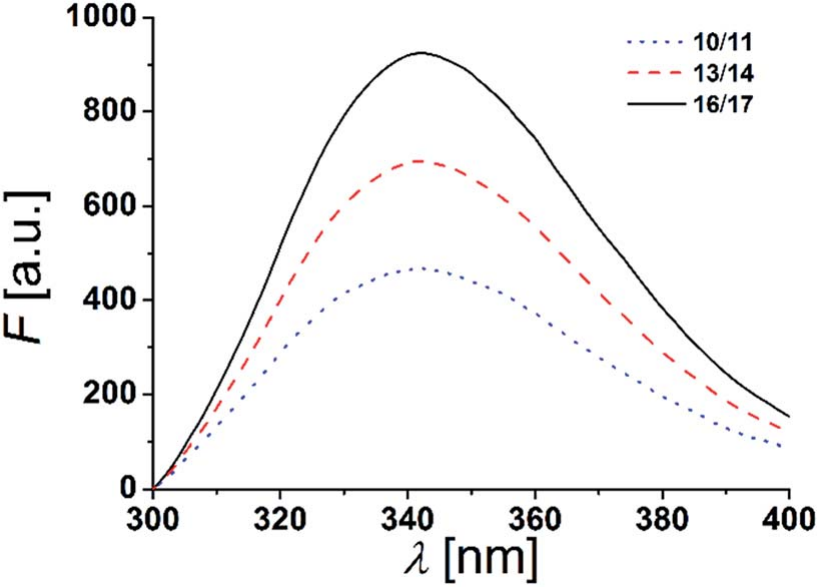
Fluorescence spectra of equimolar mixtures of **10**/**11**, **13**/**14** and **16**/**17** in DOPC LUVs (peptide concentration: 12 μm, P/L-ratio = 1/500, 25 °C).

The fluorescence emission spectra of equimolar mixtures of the β-peptides **10**/**11**, **13**/**14** and **16**/**17** in DOPC (LUVs, peptide concentration: 12 μm, P/L-ratio = 1/500, 25 °C) are shown in Fig. 5. In all three cases, the fluorescence emission *λ*_max_ of the tryptophan residues was determined to be 342 nm. If the tryptophan residues were located in a hydrophobic environment like the inner membrane part, *λ*_max_ would be expected to be < 330 nm due to interactions of the indole ring with the acyl chains of the lipids.^13^,^45^,^46^ The tryptophan emission is, however red-shifted (*λ*_max_ > 330 nm), if the tryptophan residues are located in a more polar environment.^13^ Water exposure of the indole ring results in a fluorescence maximum at 350 nm.^47^ From the obtained *λ*_max_ = 342 nm, we conclude that the tryptophan residues are located in a polar environment, *i.e.* at the polar/apolar interface of the lipid bilayer. This suggests that the β-peptides are orientated in a transmembrane fashion within the DOPC bilayer.

To conclude, the synthesized β-peptides form a stable secondary structure in the membrane environment with a CD-signature characteristic for a right-handed 3_14_-helix.^22^,^28^,^36^ From X-ray crystallography data it is known that an ideal 3_14_-helix has three amino acid residues per turn with a pitch of 5.0 Å.^48^d-β^3^-Tryptophan is located at the polar/apolar interface as determined by the intrinsic tryptophan fluorescence, which is in agreement with our recently published X-ray diffraction data obtained for very similar transmembrane β-peptides.^49^ The X-ray analysis clearly demonstrate that the peptides insert in a lipid bilayer in a transmembrane fashion, with the ^h^Trp localized at the lipid–water interface.^49^ Consequently, the d-β^3^-lysine residues are expected to be localized in the aqueous environment. Assuming an ideal 3_14_-helical structure, the membrane incorporated part of the β-peptide helix would have a length of 38.3 Å. The thickness of a DOPC bilayer, given by the distance between the phosphate groups (*D*_HH_) resulting from X-ray studies, is *D*_HH_ = 36.7 Å.^50^ Hence, the transmembrane part of the β-peptides is expected to be slightly longer than the thickness of the DOPC bilayer, which would result in a helical tilt angle of ∼16°, which was confirmed by the recently performed X-ray studies.^49^

### Determination of peptide aggregation state using FRET

**Fig. 6:**
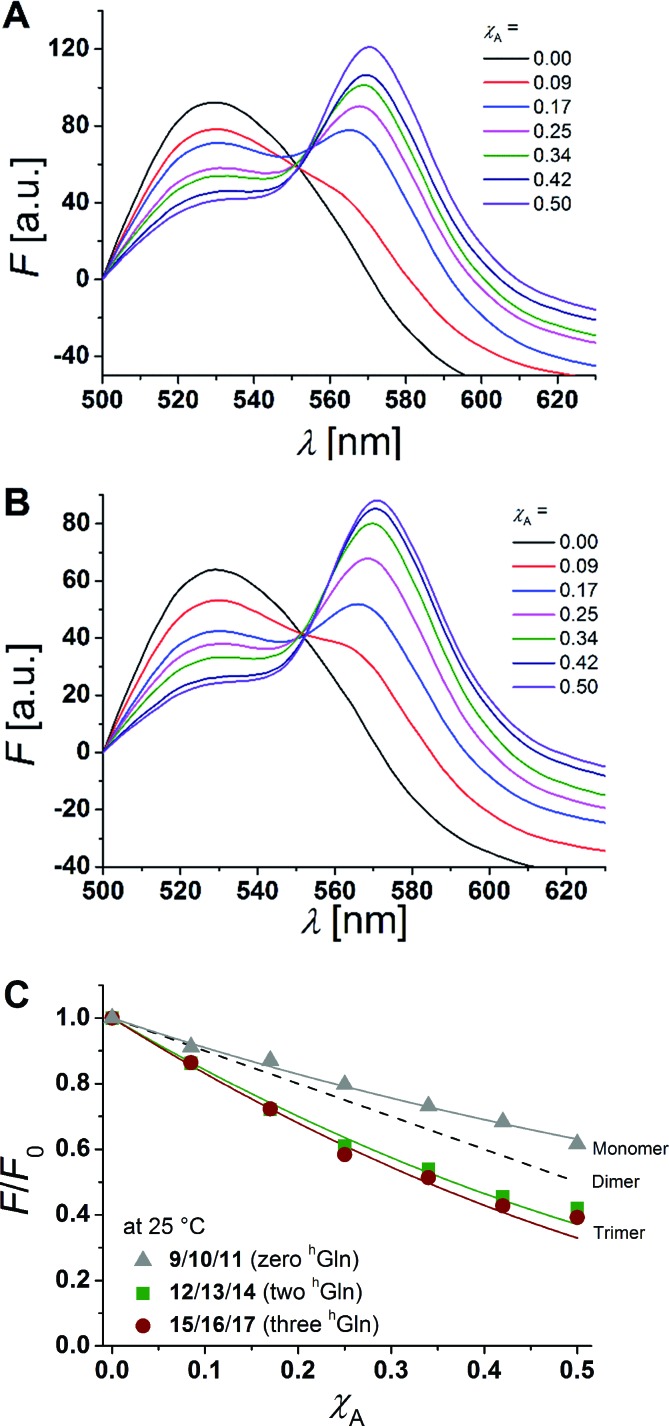
(A + B) Fluorescence emission spectra of NBD-labelled β-peptides (donor, 6.0 μm) and varying amounts of TAMRA-labelled species from *χ*_A_ = 0–0.5 determined at 25 °C. The non-labelled compound was added to keep the total peptide concentration constant (12 μm) and the P/L-ratio at 1/500 (DOPC). (A) **12**/**13**/**14** with two ^h^Gln and (B) **15**/**16**/**17** with three ^h^Gln. (C) Relative changes in NBD-fluorescence emission (*F*/*F*_0_) as a function of increasing acceptor concentration (*χ*_A_) are plotted for all three cases at 25 °C (**9**/**10**/**11** with zero ^h^Gln, **12**/**13**/**14** with two ^h^Gln and **15**/**16**/**17** with three ^h^Gln). The grey solid line is the result of a model according to Wolber *et al.*, by taking only statistical occurrence of FRET in vesicles without the formation of aggregates into account.^6^,^53^ A monomer–dimer equilibrium does not explain the data. Even the assumption of a pure dimer (dashed black line) does not explain the observed plots. The solid lines are results of the global fit analysis, which takes a monomer–trimer equilibrium into account with *K*_D_ = (17.2 ± 7.0) × 10^–8^ MF^2^ (two ^h^Gln) and *K*_D_ = (4.4 ± 4.3) × 10^–8^ MF^2^ (three ^h^Gln).

^h^Gln residues were investigated as potential recognition units within the membrane in order to control the association state of the β-peptides reconstituted into DOPC vesicles. To analyse the aggregation state, we made use of fluorescence resonance energy transfer (FRET). Therefore, β-peptides were labelled with a donor- (NBD)–acceptor- (TAMRA) pair (Fig. 3).^51^,^52^ Fig. 6A and B show fluorescence emission spectra of the β-peptides **12**/**13**/**14** (two ^h^Gln, Fig. 6A) and **15**/**16**/**17** (three ^h^Gln, Fig. 6B) at a P/L-ratio of 1/500 at 25 °C (see ESI for all fluorescence emission spectra, Fig. S4–S8†).

**Fig. 7:**
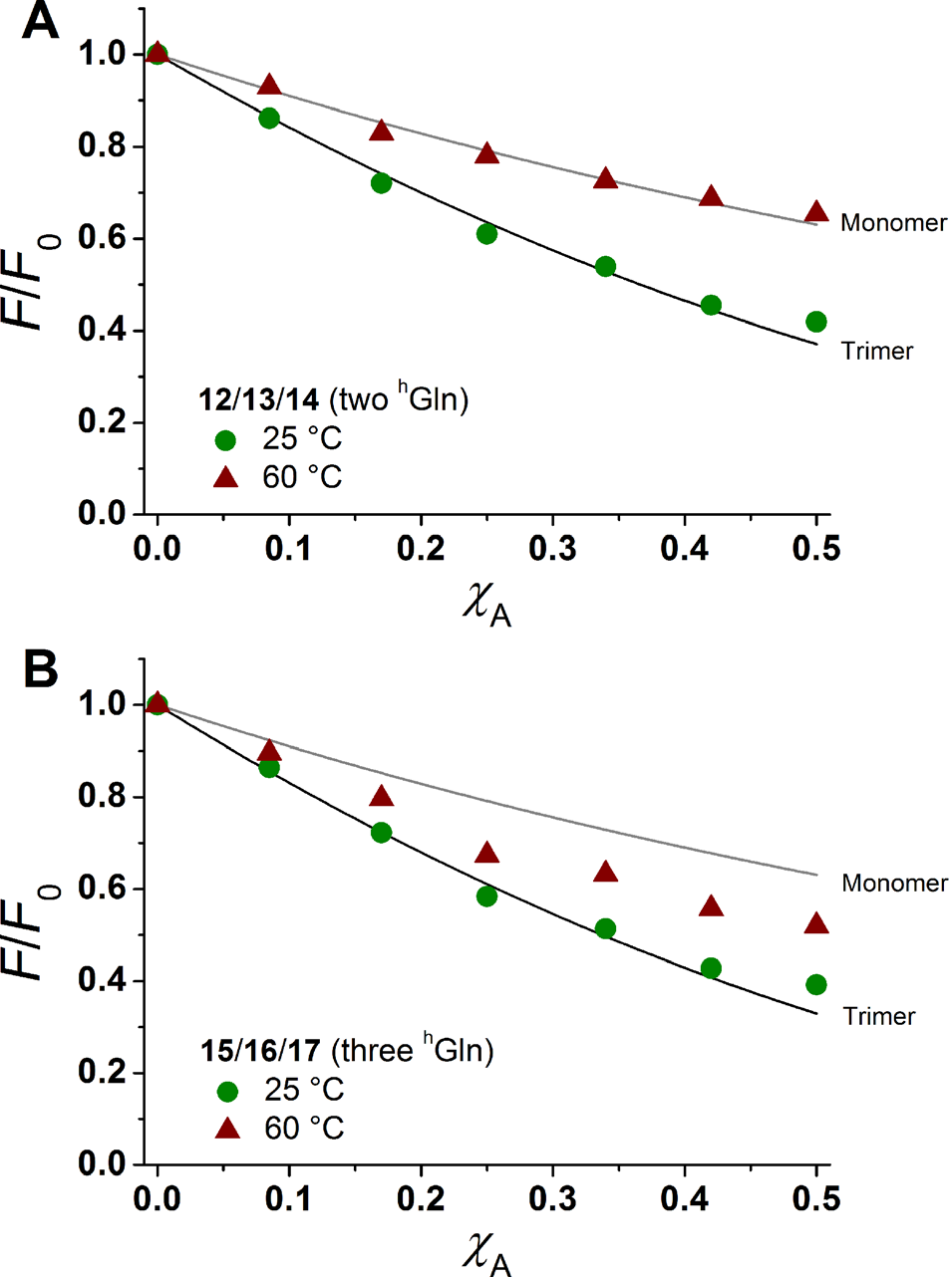
Relative changes in NBD-fluorescence emission (*F*/*F*_0_) as a function of increasing acceptor concentration (*χ*_A_) are plotted for **12**/**13**/**14** with two ^h^Gln (A) and **15**/**16**/**17** with three ^h^Gln (B) as recognition units at 25 °C and 60 °C. The grey solid line is the result of a model according to Wolber *et al.*, taking only statistical occurrence of FRET in vesicles without the formation of aggregates into account.^6^,^53^ The black solid lines are the results of a global fit analysis which takes a monomer–trimer equilibrium into account with *K*_D_ = (17.2 ± 7.0) × 10^–8^ MF^2^ (two ^h^Gln) and *K*_D_ = (4.4 ± 4.3) × 10^–8^ MF^2^ (three ^h^Gln).

The degree of β-peptide aggregation becomes accessible by measuring the ratio of NBD-fluorescence intensity *F* at 530 nm in the presence of TAMRA-labelled β-peptides and *F*_0_, the NBD fluorescence intensity at 530 nm in its absence as a function of the TAMRA-labelled β-peptide concentration, while keeping the total peptide/lipid-ratio constant by adding the acetylated non-labelled β-peptide (Fig. 6A and B).^15^ It is expected that the β-peptide lacking ^h^Gln residues remains monomeric in lipid vesicles, while the β-peptides with two or three ^h^Gln residues tend to aggregate as a result of hydrogen bond formation in the membrane. The ratio *F*/*F*_0_ as a function of the mole fraction (*χ*_A_) of the acceptor of the β-peptides at 25 °C for all described cases (zero ^h^Gln, two ^h^Gln and three ^h^Gln) are shown in Fig. 6C. In all cases, *F*/*F*_0_ decreases with increasing mole fraction (*χ*_A_) of the acceptor of the β-peptides. However, for the β-peptides with two ^h^Gln and three ^h^Gln, the decrease is significantly enhanced compared to that without ^h^Gln indicating a difference in the association behaviour of the three β-peptides. Assuming that the β-peptides without ^h^Gln (**9**/**10**/**11**) are monomeric in vesicles, the statistically occurring FRET in the vesicles can be calculated according to Wolber *et al.* taking into account an area of 0.7 nm^2^ for a DOPC lipid, a P/L ratio of 1/500 and a vesicle diameter of 100 nm.^6^,^53^ The grey solid line (Fig. 6C) is the result of a fitting routine, from which a Förster radius of *R*_0_ = 5.1 ± 0.1 nm was determined. This value is in excellent agreement with previously obtained Förster radii confirming our notion that the β-peptides without ^h^Gln are monomeric.^6^ For the two other β-peptide cases, a more pronounced FRET was observed, which indicates that the β-peptides indeed oligomerize. We analysed the data sets obtained for different P/L-ratios (1/500, 1/750, and 1/1000, ESI Fig. S7–S9†) assuming a monomer–dimer and a monomer–trimer equilibrium (Fig. 6C).^6^,^15^,^16^,^53^ No agreement between the data and the global fit analysis were found for a monomer–dimer equilibrium. Even the assumption of a pure dimer (Fig. 6C, dashed black line) does not explain the observed plots. Taking a monomer–trimer equilibrium into account, the data are in agreement between the fit with dissociation constants *K*_D_ = (17.2 ± 7.0) × 10^–8^ MF^2^ for β-peptides with two ^h^Gln (**12**/**13**/**14**) and *K*_D_ = (4.4 ± 4.3) × 10^–8^ MF^2^ for β-peptides with three ^h^Gln (**15**/**16**/**17**) (Fig. 6C, solid lines). The results provide strong evidence that the β-peptides **12**/**13**/**14** (two ^h^Gln) and **15**/**16**/**17** (three ^h^Gln) form aggregates as a result of hydrogen bond formation owing to the ^h^Gln residues. To investigate the impact of hydrogen bond formation on the aggregation properties in more detail, the temperature was varied. Hydrogen bonds are known to become weaker at higher temperatures.^54^,^55^ CD-spectroscopic measurements of the β-peptides **12** and **15** at 25 °C and at 60 °C have shown that the synthesized β-peptides form very stable secondary structures, even at higher temperature. Thus, if hydrogen bonds are the major driving force for aggregate formation, an increase in temperature should influence the aggregation state of the β-peptide helices. Fluorescence emission spectra of the β-peptides **9**/**10**/**11**, **12**/**13**/**14** and **15**/**16**/**17** within lipid bilayers (P/L-ratio = 1/500) were measured at 25 °C and 60 °C (ESI **9**/**10**/**11** in Fig. S4† at 25 °C, **12**/**13**/**14** in Fig. S5† at 25 °C and 60 °C, and **15**/**16**/**17** in Fig. S6† at 25 °C and 60 °C). Since the ratio *F*/*F*_0_ is measured, any temperature effects such as the temperature dependency of the extinction coefficients of the fluorophores NBD and TAMRA are ruled out.^6^Fig. 7 shows the results obtained at 25 °C and 60 °C together with the expected *F*/*F*_0_ (*χ*_A_) for monomeric β-peptides. It is obvious that for both β-peptide species, the observed FRET is reduced at 60 °C compared to that at 25 °C. In case of **12**/**13**/**14** (two ^h^Gln), the β-peptides appear to be in the monomeric state at 60 °C (Fig. 7A), while for **15**/**16**/**17** (three ^h^Gln), the β-peptides do not fully dissociate into monomers, but still show an altered monomer–trimer equilibrium (Fig. 7B). As these β-peptides have one additional recognition unit compared to **12**/**13**/**14**, the full dissociation does not occur, even at 60 °C owing to a larger number of hydrogen bonds in the aggregates. These results strongly support our notion that hydrogen bond formation *via* the d-β^3^-glutamine residues allows to control the aggregation state of the β-peptides in a membrane environment.

## Conclusion

The organization and assembly of transmembrane peptide helices in lipid membranes is pivotal for proper function and is influenced by a number of different factors such as helix–helix and lipid–helix interactions. For transmembrane β-peptides with a defined secondary structure, these factors are barely understood and the control of helix–helix interaction in lipid bilayers has not yet been reported. We successfully designed, synthesized and reconstituted transmembrane β-peptides, modified with d-β^3^-glutamine as a specific recognition unit to elucidate the impact of hydrogen bond formation within the lipid bilayer on helix–helix recognition and assembly. By varying the number of ^h^Gln in the helix, we were able to tune the aggregation state (monomeric or oligomeric) and the strength of the helix–helix interaction. Owing to its similarity with the well-defined 3_14_-helix where every third amino acid residue is oriented on the same face of the helix, a specific labelling of the transmembrane β-peptides makes it readily possible to precisely position a number of recognition units and hence tune the aggregation state and the strength of the interaction. This will open the avenue to rationally design different β-peptide assemblies with different functionalities.

## Experimental

### Syntheses of the d-β^3^-amino acid residues and the β-peptides

The d-β^3^-amino acids derivatives **1–7** were synthesized in excellent yields by an optimized Arndt–Eistert homologation method and were used without further purification.^48^,^56^,^57^ The synthesis of aromatic β^3^-amino acids is prone to racemization.^58^ Thus, the enantiomeric purity of Fmoc-d-β^3^-Trp(Boc)-OH (**6**) was proven using Marfeys reagent (Fig. S1†).^59^ The β-peptides **9–17** were synthesized using manual microwave-assisted Fmoc-solid phase peptide synthesis (SPPS).^60^,^61^ ^h^Lys, ^h^Trp and ^h^Gln were used with orthogonally protected side chains to avoid side reactions. To attach the fluorophores 5(6)-carboxytetra-methylrhodamine (5(6)-TAMRA) and 4-chloro-7-nitrobenzo-2-oxa-1,3-diazol (NBD-Cl) to the respective β-peptide, orthogonally protected ^h^Lys were used. After Fmoc-deprotection, NBD-Cl was linked to the side chain of Boc-d-β^3^-Lys(Fmoc)-OH (**2**), which was coupled as final d-β^3^-amino acid to the N-terminus of the β-peptide. Fmoc-d-β^3^-Lys(Alloc)-OH (**3**) was introduced at the C-terminus of the β-peptide sequence providing orthogonality to the Boc-protecting group. However, during Alloc-deprotection the terminal Fmoc-group was also deprotected. Thus, Boc-d-β^3^-Lys(Boc)-OH (**8**) was coupled to the N-terminal end of the β-peptide and after Alloc-deprotection, 5(6)-TAMRA was attached to the β-peptide sequence. All experimental procedures are described in detail in the ESI.†

### Peptide reconstitution in large unilamellar vesicles

Large unilamellar vesicles (LUVs) of 1,2-dioleoyl-*sn*-glycero-3-phosphocholine (DOPC) were prepared in TRIS® buffer (100 mm NaCl, 25 mm TRIS, pH = 7.1).^62^ Lipids were dissolved in chloroform and β-peptides in methanol. The β-peptide concentrations of the stock solutions were determined by UV absorption spectroscopy. Lipids and β-peptides were added in a test tube in the required concentrations to adjust the peptide/lipid-ratio (P/L-ratio). Removing the solvent in a nitrogen stream at room temperature produced almost clear peptide/lipid films at the test tube walls. To induce secondary structure formation the peptide/lipid films were treated with trifluoroethanol.^35^,^50^ Solvent was again removed in a nitrogen stream and the films were dried under reduced pressure at 40 °C overnight. Multilamellar vesicles were produced by hydration of the peptide/lipid film in TRIS buffer. After 2 h of incubation at 25 °C the hydrated peptide/lipid films were vortexed (30 s) and incubated (5 min) in five cycles. The milky suspensions were extruded 31 times through a polycarbonate membrane with 100 nm nominal pore size using the Avestin Liposofast mini extruder (Ottawa, Canada) to obtain clear vesicle suspensions. In case of fluorescence spectroscopy, DOPC dissolved in chloroform and the β-peptides **9–17** dissolved in methanol were mixed in the order: (i) DOPC, (ii) NBD labelled β-peptide (**10**, **13**, **16**), (iii) TAMRA labelled β-peptide (**11**, **14**, **17**), (iv) non-labelled β-peptide (**9**, **12**, **15**). For CD-spectroscopic analysis, the β-peptides **9**, **12** and **15** dissolved in methanol were mixed with DOPC dissolved in chloroform.

### Circular dichroism (CD) spectroscopy

All CD-spectra were measured on a Jasco-1500 spectropolarimeter (Goβ-Umstadt, Germany) equipped with a Julabo F250 (Seelbach, Germany) temperature control unit and recorded at 25 °C and at 60 °C in a wavelength range of 260–180 nm. The CD-spectra were background-corrected against pure vesicle suspension without incorporated β-peptides or TFE and expressed as molar ellipticity *θ* (deg cm^2^ dmol^–1^), according to Greenfield.^63^

### Tryptophan fluorescence and fluorescence resonance energy transfer (FRET)

All fluorescence spectra were measured on a Jasco FP 6200 (Groβ-Umstadt, Germany) under temperature control using a Jasco thermostat (model ETC-272T, Groβ-Umstadt, Germany). Tryptophan fluorescence of the β-peptides was excited at 280 nm and the emission detected in the range of 300–400 nm. The association of the synthesized β-peptides in lipid bilayers was investigated by FRET. The β-peptides **10**, **13** and **16** were labelled with NBD as donor fluorophore and TAMRA served as acceptor in the β-peptides **11**, **14** and **17**. The concentration of the respective NBD-equipped β-peptide (6.0 μm) as well as the total β-peptide concentration (12 μm) were kept constant using the corresponding non-labelled β-peptide **9**, **12** or **15**. The mole fraction (*χ*_A_) of the respective TAMRA-labelled β-peptide was varied from 0–0.5. After extrusion of the lipid vesicles (DOPC, LUVs, P/L-ratio = 1/500), the fluorescence emission of the samples was measured at 25 °C and 60 °C. The fluorescence was excited at 464 nm and the fluorescence emission was monitored between 500 and 650 nm. The theoretical treatment of the obtained FRET data was performed as described previously.^15^,^16^,^52^,^53^,^64^,^65^

## Supplementary Material

Supplementary information
