# Supplementary material for: β-Glutamine-mediated self-association of transmembrane β-peptides within lipid bilayers
Source: Chem Sci. 2016 May 19;7(9):5900–7. doi: 10.1039/c6sc01147k (PMC6022121; doi:10.1039/c6sc01147k)
Supplement: Supplementary file 1 [file SC-007-C6SC01147K-s001.pdf]

Electronic Supplementary Material (ESI) for Chemical Science.
This journal is © The Royal Society of Chemistry 2016

β-Glutamine-mediated Self-Association of Transmembrane β-Peptides within Lipid Bilayers

U. Rost, C. Steinem, U. Diederichsen**

Institute for Organic and Biomolecular Chemistry, Georg-August-University Göttingen, Tammannstr. 2, 37077 Göttingen, Germany

Email: csteine@gwdg.de; udieder@gwdg.de

Materials and Methods

Solvents: Dry THF was purchased from ACROS ORGANICS (Geel, Belgium) and stored over molecular sieves (4 Å). Solvents of analytical or HPLC grade were used as supplied from FLUKA (Taufkirchen, Germany), VWR INTERNATIONAL (Fontenay-sous-Bois, France), ACROS ORGANICS (Geel, Belgium) and SIGMA-ALDRICH (Taufkirchen, Germany). Ultrapure water was obtained using the water purification unit SIMPLICITY (MILLIPORE, Bredford, UK). **Reagents:** All reagents were of highest grade available and used as supplied. All amino acid derivatives as well as coupling reagents and the resin for solid phase peptide synthesis (SPPS) were obtained from NOVABIOCHEM (Darmstadt, Germany), BACHEM (Bubendorf, Switzerland), GL BIOCHEM (Shanghai, China), IRIS BIOTECH (Marktredwitz, Germany) and VWR INTERNATIONAL (Fontenay-sous-Bois, France). DOPC was purchased from AVANTI POLAR LIPIDS (Alabama, USA). All other chemicals were purchased from FLUKA (Taufkirchen, Germany), VWR INTERNATIONAL (Fontenay-sous-Bois, France), ACROS ORGANICS (Geel, Belgium), SIGMA-ALDRICH (Taufkirchen, Germany), ALFA AESAR (Karlsruhe, Germany), APPLICHEM (Darmstadt, Germany), TCI (Zwijndrecht, Belgium), ABCR (Karlsruhe, Germany), FLUOROCHEM (Hadfield, UK), CARL ROTH GMBH (Karlsruhe, Germany), GRÜSSING GMBH (Filsum, Germany), MERCK (Darmstadt, Germany) and RIEDEL-DE HAËN (Seelze, Germany). **Reactions:** Glass equipment utilized for reactions under inert atmosphere was heated under reduced pressure and flushed with argon prior to use (SCHLENK-technique). **HPLC:** Purification and analysis was performed using AMERSHAM PHARMACIA BIOTECH (Freiburg, Germany) (ÄKTA BASIC 900, pump type P 900, variable wavelength detector UV-900). The UV-absorption was detected at 215 nm, 254 nm and 280 nm for non-labelled peptides and for derivatives labelled with NBD or TAMRA the UV-absorption was recorded at 464 nm or 540 nm applying a linear gradient of A (water + 0.1% TFA) to B (MeOH + 0.1% TFA). **Analysis:** NMR spectra were recorded on a VARIAN spectrometer (Unity 300 or Inova 500) at 35 °C ([D6]DMSO). ESI-MS and HR-MS (ESI) spectra were obtained with BRUKER devices (maXis or MicrOTOF). **UV-Spectroscopy:** UV spectra for estimation of occupancy or peptide concentration were measured using the THERMO SCIENTIFIC Nanodrop ND-2000c spectrophoto-meter (Munich, Germany). Peptide concentration was calculated using LAMBERT-BEER's law (for extinction coefficients see Table S1). The molecular absorption coefficients were calculated by summation of the single coefficients at a set wavelength and for labelled molecules the absorption was measured at the maximum absorption of the labels (NBD, TAMRA).

Table S1: Extinction coefficients for tryptophan (Trp), NBD and TAMRA. [1,2]

	Absorption Maximum [nm]	Extinction Coefficient [cm^{-1}M^{-1}]
Trp	280	5690
NBD	466	22000
TAMRA	565	91000

CD-Spectroscopy: CD spectra were measured on a JASCO-1500 spectropolarimeter (Groß-Umstadt, Germany) equipped with a JULABO F250 (Seelbach, Germany) temperature control unit. The sample cell was flushed with nitrogen and a quartz glass precision cell Suprasil® (type: 110-QS, 1 mm) was used. The spectra were recorded at 25 °C or 60 °C in a wavelength range of 260-180 nm with 1.0 nm bandwidth, using the ´continuous mode´, a response time of 1.0 s, a scanning speed of 20 nm/min, a data pitch of 1.0 nm and an average of five spectra. The spectra were background-corrected against pure vesicle suspension without incorporated peptides or TFE, smoothed (SAVITZKY-GOLAY) and expressed as molar ellipticity θ (deg cm^2 dmol^{-1}), according to GREENFIELD.[3] **Fluorescence spectroscopy:** Fluorescence spectra were obtained at a JASCO FP 6200 (Groß-Umstadt, Germany) under temperature control using a JASCO thermostat (model ETC-272T, Groß-Umstadt, Germany). For analysing the tryptophan fluorescence emission, excitation was performed at 280 nm and the fluorescence emission was detected between 290-420 nm. In case of the concentration-dependent FRET analysis, the fluorescence emission was detected between 500-650 nm at 25 °C and 60 °C for the whole sample set and the excitation was performed at 464 nm. A micro quartz glass precision cell Suprasil® (type: 115F-QS, 10 mm) was used and the excitation- and emission-bandwidths were set to 5 nm, the data pitch was 1 nm, the response fast, the sensitivity high and the scanning speed was set to 125 nm/min.

Syntheses of the D-β^3-amino acids[4–6]

To optimize the procedure of GUICHARD *et al.*, under an argon atmosphere the Boc/Fmoc-protected amino acid (1.00 eq) was dissolved in dry THF (4.5 mL/mmol) and cooled to 0 °C, followed by addition of triethylamine (1.10 eq) and isobutylchloroformate (1.10 eq). After stirring

at 0 °C for 30 min, under exclusion of light diazomethane (0.6-0.7 M in diethyl ether, 2.00 eq) was added to the solution and the mixture was stirred at rt for 5 h. Afterwards, the excess of diazomethane was quenched by addition of glacial acetic acid (0.03 eq) and the mixture was taken up in 6% $NaHCO_3$ (aq, 8.0 mL/mmol) and extracted with EtOAc (3 ×). The combined organic phases were washed with saturated NH_4Cl (aq, 3 ×), saturated NaCl (aq, 3 ×), dried over $MgSO_4$ and the solvent was removed under reduced pressure. The product (yellow oil/solid) was obtained in quantitative yield and used without further purification. The resulting diazo ketone (1.00 eq) was dissolved in THF/H_2O (9:1, 6.0 mL/mmol) and under exclusion of light silver benzoate (0.10 eq) was added. The reaction was carried out by sonication for 2 h. Afterwards, the mixture was diluted with H_2O (4.0 mL/mmol), adjusted to pH = 2-3 with 1.0 M HCl and extracted with EtOAc (3 ×). The combined organic phases were dried over $MgSO_4$ and the solvent was removed under reduced pressure. Alternatively, by optimizing the procedure of PATIL *et al.*, silver benzoate (0.10 eq) was added to a solution of the diazo ketone (1.00 eq) in 1,4-dioxane/H_2O (2:1, 10 mL/mmol) and the reaction was performed under microwave irradiation in a domestic microwave oven (55 s, 460 W). Afterwards, the solvent was removed under reduced pressure and the resulting residue was diluted with H_2O (3.5 mL/mmol). The mixture was adjusted to pH = 2-3 with 1.0 M HCl and extracted with EtOAc (3 ×). The combined organic phases were dried over $MgSO_4$ and the solvent was removed under reduced pressure. Finally, in both cases the crude product was taken up in DCM and added dropwise to cold pentane (-22 °C). The obtained precipitate was filtered off, washed with cold pentane (2 ×) and dried overnight under reduced pressure (Table S2).

Table S2: Synthesized D-β^3-amino acids and the obtained yield over two steps; [a]sonication; [b]domestic microwave.

D-β^3-amino acid	Yield in %
Fmoc-D-β^3-Lys(Boc)-OH (**1**)	83[b]
Boc- D-β^3-Lys(Fmoc)-OH (**2**)	72[a]
Fmoc- D-β^3-Lys(Alloc)-OH (**3**)	70[a]
Fmoc- D-β^3-Val-OH (**4**)	94[b]
Fmoc- D-β^3-Trp(Boc)-OH (**5**)	75[a]
Fmoc- D-β^3-Gln(Trt)-OH (**6**)	72[a]
Fmoc-D/L- β^3-Trp(Boc)-OH (**7**)	68[a]

Analytical data of the synthesized D-β^3-amino acids

(*R*)-7-*tert*-Butoxycarbonylamino-3-(9-fluorenylmethyloxycarbonylamino)-heptanoic acid (1)
The product was obtained as a colourless solid. **^{1}H-NMR** (300 MHz, [D6]DMSO): δ (ppm) = 1.12-1.46 (m, 15 H, 3 × CH_3, γ-CH_2, δ-CH_2, ε-CH_2), 2.26-2.40 (m, 2 H, ζ-CH_2), 2.82-2.92 (m, 2 H, α-CH_2), 3.68-3.83 (m, 1 H, β-CH), 4.17-4.32 (m, 3 H, Fmoc-CH_2, Fmoc-CH), 6.69 (d, 1 H, $^3J_{H,H}$ = 5.9 Hz, NH), 7.15 (d, 1 H, $^3J_{H,H}$ = 8.6 Hz, NH), 7.28-7.45 (m, 4 H, 4 × Fmoc-CH_{ar}), 7.68 (d, 2 H, $^3J_{H,H}$ = 7.5 Hz, 2 × Fmoc-CH_{ar}), 7.88 (d, 2 H, $^3J_{H,H}$ = 7.5 Hz, 2 × Fmoc-CH_{ar}), 12.1 (s_{br}, 1 H, OH). **ESI-MS** (*m/z*): 483.3 $[M+H]^+$, 505.3 $[M+Na]^+$, 521.2 $[M+K]^+$, 987.5 $[2M+Na]^+$, 1003.5 $[2M+K]^+$. **HR-MS** (ESI): calc. for $[C_{27}H_{35}N_2O_6]^+$ ($[M+H]^+$): 483.2490, found: 483.2476; calc. for $[C_{27}H_{34}N_2O_6Na]^+$ ($[M+Na]^+$): 505.2309, found: 505.2309; calc. for $[C_{27}H_{34}N_2O_6K]^+$ ($[M+K]^+$): 521.2048, found: 521.2056.

(*R*)-3-*tert*-Butoxycarbonylamino-7-(9-fluorenylmethyloxycarbonylamino)-heptanoic acid (2)
The product was obtained as a colourless solid. **^{1}H-NMR** (300 MHz, [D6]DMSO): δ (ppm) = 1.17-1.47 (m, 15 H, 3 × CH_3, γ-CH_2, δ-CH_2, ε-CH_2), 2.24-2.37 (m, 2 H, ζ-CH_2), 2.91-3.02 (m, 2 H, α-CH_2), 3.66-3.77 (m, 1 H, β-CH), 4.15-4.36 (m, 3 H, Fmoc-CH_2, Fmoc-CH), 6.60 (d, 1 H, $^3J_{H,H}$ = 8.7 Hz, NH), 7.20 (t, 1 H, $^3J_{H,H}$ = 5.7 Hz, NH), 7.28-7.45 (m, 4 H, 4 × Fmoc-CH_{ar}), 7.68 (d, 2 H, $^3J_{H,H}$ = 7.4 Hz, 2 × Fmoc-CH_{ar}), 7.87 (d, 2 H, $^3J_{H,H}$ = 7.5 Hz, 2 × Fmoc-CH_{ar}), 12.1 (s_{br}, 1 H, OH). **ESI-MS** (*m/z*): 483.3

[M+H]$^+$, 505.3 [M+Na]$^+$, 521.2 [M+K]$^+$, 987.5 [2M+Na]$^+$. **HR-MS** (ESI): calc. for $[C_{27}H_{34}N_2O_6Na]^+$ ([M+Na]$^+$): 505.2309, found: 505.2309.

(*R*)-7-Allyloxycarbonylamino-3-(9-fluorenylmethyloxycarbonylamino)-heptanoic acid (3) The product was obtained as a colourless solid. **^{1}H-NMR** (300 MHz, [D6]DMSO): δ (ppm) = 1.11-1.52 (m, 6 H, γ-CH_2, δ-CH_2, ε-CH_2), 2.21-2.44 (m, 1 H, ζ-CH_2), 2.87-3.03 (m, 2 H, α-CH_2), 3.69-3.85 (m, 1 H, β-CH), 4.14-4.36 (m, 3 H, Fmoc-CH_2, Fmoc-CH), 4.37-4.54 (m, 2 H, Alloc-CH_2), 5.09-5.32 (m, 2 H, Alloc-CH_{2allyl}), 5.89 (ddt, 1 H, $^3J_{HH}$ = 17.3, 10.6, 5.3 Hz, Alloc-CH_{allyl}), 7.05-7.20 (m, 2 H, 2 × NH), 7.27-7.45 (m, 4 H, 4 × Fmoc-CH_{ar}), 7.68 (d, 2 H, $^3J_{H,H}$ = 7.4 Hz, 2 × Fmoc-CH_{ar}), 7.88 (d, 2 H, $^3J_{H,H}$ = 7.4 Hz, 2 × Fmoc-CH_{ar}), 12.1 (s_{br}, 1 H, OH). **ESI-MS** (*m/z*): 467.2 [M+H]$^+$, 489.2 [M+Na]$^+$, 955.4 [2M+Na]$^+$. **HR-MS** (ESI): calc. for $[C_{26}H_{31}N_2O_6]^+$ ([M+H]$^+$): 467.2177, found: 467.2170; calc. for $[C_{26}H_{30}N_2O_6Na]^+$ ([M+Na]$^+$): 489.1996, found: 489.2003.

(*R*)-3-(9-Fluorenylmethyloxycarbonylamino)-4-methyl-pentanoic acid (4) The product was obtained as a colourless solid. **^{1}H-NMR** (300 MHz, [D6]DMSO): δ (ppm) = 0.59-0.88 (m, 6 H, 2 × CH_3), 1.59-1.80 (m, 1 H, γ-CH), 2.21-2.46 (m, 2 H, α-CH_2), 3.56-3.83 (m, 1 H, β-CH), 4.07-4.38 (m, 3 H, Fmoc-CH_2, Fmoc-CH), 7.16 (d, 1 H, $^3J_{H,H}$ = 9.0 Hz, NH), 7.26-7.43 (m, 4 H, 4 × Fmoc-CH_{ar}), 7.64-7-71 (m, 2 H, 2 × Fmoc-CH_{ar}), 7.86 (d, 2 H, $^3J_{H,H}$ = 7.6 Hz, 2 × Fmoc-CH_{ar}), 12.0 (s_{br}, 1 H, OH). **ESI-MS** (*m/z*): 354.2 [M+H]$^+$, 376.2 [M+Na]$^+$, 392.1 [M+K]$^+$, 707.4 [2M+H]$^+$, 729.3 [2M+Na]$^+$, 745.3 [2M+K]$^+$, 352.2 [M-H]$^-$, 705.3 [2M-H]$^-$. **HR-MS** (ESI): calc. for $[C_{21}H_{24}NO_4]^+$ ([M+H]$^+$): 354.1700, found: 354.1698; calc. for $[C_{21}H_{23}NO_4Na]^+$ ([M+Na]$^+$): 376.1519, found: 376.1519; calc. for $[C_{21}H_{22}NO_4]^-$ ([M-H]$^-$): 352.1554, found: 352.1555.

(*R*)-4-(1`-(2`-*tert*-Butoxycarbonyl)-indol-3`-yl)-3-(9-fluorenylmethyloxycarbonylamino)-butanoic acid (5, 7) The product was obtained as a yellowish solid. **^{1}H-NMR** (300 MHz, [D6]DMSO): δ (ppm) = 1.55 (s, 9 H, 3 × CH_3), 2.44-2.51 (m, 2 H, γ-CH_2), 2.87 (d, 2 H, $^3J_{H,H}$ = 6.7 Hz, α-CH_2), 4.07-4.28 (m, 4 H, β-CH, Fmoc-CH_2, Fmoc-CH), 7.19-7.44 (m, 7 H, NH, 4 × Fmoc-CH_{ar}, 2 × CH_{ar}), 7.51 (s, 1 H, CH_{ar}), 7.54-7.72 (m, 2 H, 2 × Fmoc-CH_{ar}), 7.70 (d, 1 H, $^3J_{H,H}$ = 7.7 Hz, CH_{ar}), 7.86 (d, 2 H, $^3J_{H,H}$ = 7.6 Hz, 2 × Fmoc-CH_{ar}), 8.03 (d, 1 H, $^3J_{H,H}$ = 8.1 Hz, CH_{ar}), 12.2 (s_{br}, 1 H, OH). **ESI-MS** (*m/z*): 541.3 [M+H]$^+$, 558.3 [M+NH_4]$^+$, 563.2 [M+Na]$^+$, 1103.5 [2M+Na]$^+$, 539.2 [M-H]$^-$, 1079.4 [2M-H]$^-$. **HR-MS** (ESI): calc. for $[C_{32}H_{33}N_2O_6]^+$ ([M+H]$^+$): 541.2333, found: 541.2327; calc. for

$[C_{32}H_{32}N_2O_6Na]^+$ ($[M+Na]^+$): 563.2153, found: 563.2148; calc. for $[C_{32}H_{31}N_2O_4]^-$ ($[M-H]^-$): 539.2188, found: 539.2188.

(*R*)-3-(9-Fluorenylmethyloxycarbonylamino)-6-oxo-6-tritylamino-hexanoic acid (6) The product was obtained as a colourless solid. **^{1}H-NMR** (300 MHz, [D6]DMSO): δ (ppm) = 1.51-1.75 (m, 2 H, γ-CH_2), 2.21-2.42 (m, 4 H, α-CH_2, δ-CH_2), 3.77-3.92 (m, 1 H, β-CH), 4.18-4.35 (m, 3 H, Fmoc-CH_2, Fmoc-CH), 7.13-7.45 (m, 20 H, 3 × C_5H_5, NH, 4 × Fmoc-CH_{ar}), 7.69 (d, 2 H, $^3J_{H,H}$ = 7.4 Hz, 2 × Fmoc-CH_{ar}), 7.88 (d, 2 H, $^3J_{H,H}$ = 7.4 Hz, 2 × Fmoc-CH_{ar}), 8.49 (s, 1 H, NH), 12.1 (s_{br}, 1 H, OH). **ESI-MS** (*m/z*): 625.3 $[M+H]^+$, 647.3 $[M+Na]^+$, 1271.5 $[2M+Na]^+$, 623.3 $[M-H]^-$. **HR-MS** (ESI): calc. for $[C_{40}H_{37}N_2O_5]^+$ ($[M+H]^+$): 625.2701, found: 625.2697; calc. for $[C_{40}H_{36}N_2O_5Na]^+$ ($[M+Na]^+$): 647.2516, found: 647.2517; calc. for $[C_{40}H_{35}N_2O_5]^-$ ($[M-H]^-$): 623.2551, found: 623.2530.

Synthesis of (*R*)-3-*tert*-Butoxycarbonylamino-7-*tert*-butoxycarbonylamino-heptanoic acid (8)[7]

According to the procedure of Myers *et al.*, Fmoc-D-β^3-Lys(Boc)-OH (**1**) (1.50 g, 3.11 mmol, 1.00 eq) was dissolved in piperidine (20% in DMF, 10 mL) and stirred at rt for 15 min. Afterwards the solvent was removed under reduced pressure and the residue was taken up in diethyl ether. The solid was isolated by centrifugation (9000 rpm, 10 min, -15 °C), washed five times with diethyl ether followed by centrifugation and dried under reduced pressure. The resulting Fmoc-deprotected residue (3.11 mmol, 1.00 eq) was dissolved in H_2O (20 mL) and $NaHCO_3$ (523 mg, 6.22 mmol, 2.00 eq) was added. The reaction mixture was cooled to 0 °C, Boc_2O (813 mg, 3.73 mmol, 1.20 eq) in 1,4-dioxane (9 mL) was added dropwise at 0 °C and the mixture was stirred at rt overnight. H_2O (15 mL) was then added, the aqueous layer was extracted with EtOAc (3 × 30 mL) and the organic layers were back extracted with saturated $NaHCO_3$ (aq, 3 × 30 mL). The combined aqueous layers were adjusted to pH = 2-3 with 1.0 M HCl and extracted with EtOAc (3 × 50 mL). The combined organic phases were dried over $MgSO_4$ and the solvent was removed under reduced pressure to yield product **8** (1.03 g, 2.86 mmol, 92%) as a colourless oil. **^{1}H-NMR** (300 MHz, [D6]DMSO): δ (ppm) = 1.20-1.43 (m, 24 H, 3 × CH_3, γ-CH_2, δ-CH_2, ε-CH_2), 2.21-2.35 (m, 2 H, ζ-CH_2), 2.82-2.93 (m, 2 H, α-CH_2), 3.61-3.76 (m, 1 H, β-CH), 6.58 (d, 1 H, $^3J_{H,H}$ = 8.7 Hz, NH), 6.65 (d, 1 H, $^3J_{H,H}$ = 6.4 Hz, NH), 12.1 (s_{br}, 1 H, OH). **ESI-MS** (*m/z*): 383.2 $[M+Na]^+$, 399.2 $[M+K]^+$,

359.2 [M-H]$^-$, 719.5 [2M-H]$^-$. **HR-MS** (ESI): calc. for $[C_{17}H_{32}N_2O_6Na]^+$ ($[M+Na]^+$): 383.2153, found: 383.2151; calc. for $[C_{17}H_{32}N_2O_6K]^+$ ($[M+K]^+$): 399.1892, found: 399.1892; calc. for $[C_{17}H_{31}N_2O_6]^-$ ($[M-H]^-$): 359.2188, found: 359.2190.

Enantiomeric purity (MARFEY´s reagent)[8–10]

Fmoc-D/L-β^3-Trp(Boc)-OH (**7**) (9.65 mg, 20.0 µmol, 1.00 eq) and Fmoc-D-β^3-Trp(Boc)-OH (**5**) were treated with piperidine (20% in DMF, 5.0 mL) and stirred at rt for 15 min. After removal of the solvent under reduced pressure the corresponding residue was taken up in diethyl ether and the solid was isolated by centrifugation (9000 rpm, 10 min, 25 °C), washed five times with diethyl ether followed by centrifugation and dried under reduced pressure. According to the procedures of MAFREY *et al*. and BHUSHAN *et al*., the resulting solid was diluted in acetone/H_2O (1/1, *v*/*v*, 400 µL). 100 µL of this mixture and 0.5 M Na_2CO_3 (aq, 40.0 µL) were added to a solution of MARFEY´s reagent in acetone (1.1 mM, 200 µL). The resulting mixture was shaken for 1 h at 40 °C. Afterwards it was cooled to 2 °C, 1.0 M HCl (aq, 20.0 µL) was added and each sample was centrifuged (15000 rpm, 5 min). HPLC analysis for the D/L-mixture and D-residue was performed with a linear gradient of A (water + 0.1% TFA) to B (MeCN + 0.1% TFA) (see Fig. S1). **HPLC** (MN Nucleodur® 100-5-C18, 250 mm × 4.6 mm, 5 µm, flow rate: 1.0 mL/min, gradient: 25-70% B in 60 min, λ in nm: 280, 340).

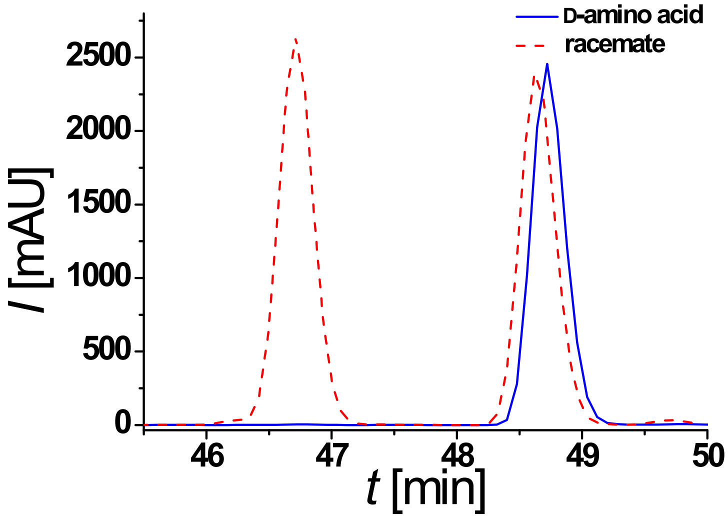

Figure S1: HPLC spectra of the D/L-mixture (red) and the D-amino acid (blue) after reaction of β^3-tryptophane with MARFEY`s reagent.

Synthesis of the β-peptides 9-17

The β-peptides were synthesized via manual Fmoc solid phase peptide synthesis (SPPS) on a rink amide MBHA resin (0.57 mmol/g) using the DISCOVER microwave (MW) reaction cavity (CEM) (Kamp-Lintfort, Germany). For the attachment of the first amino acid the resin was placed in a PE-frit equipped BECTON-DICKINSON (BD) discardit II syringe (Heidelberg, Germany) and swollen in DMF (2 mL) for 2 h. The Fmoc-protecting group was cleaved microwave assisted (25 W, 50 °C, 30 s) using piperidine in DMF (20%). Afterwards, the resin was washed with DMF (10 ×) and the second cleavage (20% piperidine in DMF) was performed (35 W, 60 °C, 3 min). After washing with DMF (10 ×), the required D-β^3-amino acid building block (5.00 eq) and HOBt (5.00 eq) were dissolved in DMF and DIC (5.00 eq) was added. The coupling was achieved using microwave irradiation (35 W, 65 °C, 15 min). The resin was washed with DMF (10 ×), MeOH (10 ×) and DCM (5 ×) before it was dried under reduced pressure overnight.[11] The estimation of the occupancy of the first residue on the resin was performed according to the procedure of GUDE *et al.* via UV-analysis of the dibenzofulven concentration resulting from Fmoc-deprotection.[12] Therefore, a small amount of resin (5-10 mg) was placed in a graduated flask (10 mL) and DBU (2% in DMF, 2.0 mL) was added. After gentle shaking for 1 h the reaction mixture was diluted with MeCN (to 10 mL). The reaction mixture was further diluted with MeCN (1/12.5) and transferred to an UV precision cuvette. The absorption of the cleaved Fmoc species was detected at 304 nm and corrected against the reference (ε_{304} = 7624 L mol^{-1} cm^{-1}). When the loading density of the resin was considered to be sufficient, the remaining free amine functions were acetylated applying DMF/Ac_2O/DIEA (18:1:1, *v*/*v*/*v*) at room temperature for 10 min, followed by washing with DMF (10 ×), MeOH (10 ×) and DCM (5 ×) and drying under reduced pressure overnight. All β-peptides were synthesized using manual SPPS which was carried out in a PE-frit equipped BECTON-DICKINSON (BD) discardit II syringe (Heidelberg, Germany) in a 75 µmol scale.[5,13] The preloaded resin initially was swollen in DMF for 2 h and the Fmoc-protecting group was cleaved as mentioned before. After washing with DMF (10 ×), the required D-β^3-amino acid building block in DMF (0.2 M, 4.0 eq) was activated with HATU/HOAt in DMF (0.46 M/0.5 M, 4.9 eq/5.0 eq) and DIPEA in NMP (2.0 M, 10 eq) was added. Single coupling was performed under microwave irritation (35 W, 65 °C, 15 min) followed by washing with DMF (10 ×). In case of the peptides **9**, **10**, **12**, **13**, **15** and **16** the following D-β^3-amino acid buildings blocks were used: Fmoc-D-β^3-Lys(Boc)-OH (**1**), Fmoc-D-β^3-

Trp(Boc)-OH (**5**), Fmoc-D-β^3-Val-OH (**4**), Fmoc-D-β^3-Gln(Trt)-OH (**6**) and Boc-D-β^3-Lys(Fmoc)-OH (**2**). After final Fmoc-deprotection, the free side chain amino functionality of the *N*-terminal ^{h}Lys was labelled with NBD-Cl (15.0 mg, 75.0 µmol, 3.00 eq) in DMF (345 µL) which was activated with DIPEA (83.0 µL, 500 µmol, 20.0 eq) and allowed to react overnight. Alternatively, the free side chain amino functionality of the *N*-terminal ^{h}Lys was acetylated with DMF/Ac_2O/DIPEA (18:1:1, *v*/*v*/*v*) at rt for 10 min. For the β-peptides **11**, **14** and **17** the following D-β^3-amino acid building blocks were used: Fmoc-D-β^3-Lys(Boc)-OH (**1**), Fmoc-D-β^3-Trp(Boc)-OH (**5**), Fmoc-D-β^3-Val-OH (**4**), Fmoc-D-β^3-Lys(Alloc)-OH (**3**), Fmoc-D-β^3-Gln(Trt)-OH (**6**) and Boc-D-β^3-Lys(Boc)-OH (**8**). Based on the procedure of WU *et al.*, the Alloc-deprotection was performed under an argon atmosphere.[14] Therefore, $Me_2NH·BH_3$ (180 mg, 3.00 mmol, 40.0 eq) and $Pd(PPh_3)_4$ (8.63 mg, 7.50 µmol, 0.10 eq) were added to the resin (75.0 µmol, 1.00 eq) in dry DMF (3 mL) and argon was bubbled through the mixture for 4 h. Afterwards, the mixture was transferred to a PE-frit equipped BECTON-DICKINSON (BD) discardit II syringe (Heidelberg, Germany) and washed with DMF (10 ×), MeOH (10 ×) and DCM (5 ×). The resulting free side chain amino functionality was labelled with 5(6)-TAMRA (53.4 mg, 125 µmol, 5.00 eq) in DMF (340 µL), which was activated with PyBOP® (61.5 mg, 118 µmol, 4.70 eq) and DIPEA (41.7 µL, 245 µmol, 9.80 eq) and allowed to react at rt overnight. After completion of the peptide sequences, washing was performed with DMF (10 x), MeOH (10 x) and DCM (10 x) and the resin was dried under reduced pressure overnight. Cleavage from the solid support and simultaneous removal of the protecting groups was carried out within 2 h using TFA/H_2O/Tis (95/2.5/2.5, *v*/*v*/*v*). The reaction mixture was concentrated under a nitrogen stream and the crude peptide was precipitated as solid using -20 °C cold diethyl ether. The respective β-peptide was isolated by centrifugation (9000 rpm, 20 min, -15 °C), washed five times with cold diethyl ether followed by centrifugation and dried under reduced pressure. HPLC purification was performed to yield the pure β-peptides **9**-**17**.

Synthesized β-peptides (analytical data)

H-^{h}Lys(acetyl)-^{h}Lys$_2$-^{h}Trp$_2$-^{h}Val$_{19}$-^{h}Trp$_2$-^{h}Lys$_2$-NH$_2$ (9)

HPLC (MN Nucleodur® 100-5-C18, 250 mm × 21 mm, 5 µm, flow rate: 10 mL/min, gradient: 88-100% B in 30 min, λ in nm: 215, 254, 280): t_R = 12.30 min. **ESI-MS** (*m*/*z*): 621.1[M+6H]$^{6+}$, 745.1 [M+5H]$^{5+}$, 931.2 [M+4H]$^{4+}$, 1241.2 [M+3H]$^{3+}$. **HR-MS** (ESI): calc. for $[C_{199}H_{335}N_{38}O_{29}]^{3+}$ ([M+3H]$^{3+}$):

1241.1984, found: 1241.1998; calc. for $[C_{199}H_{336}N_{38}O_{29}]^{4+}$ ($[M+4H]^{4+}$): 931.1506, found: 931.1514; calc. for $[C_{199}H_{337}N_{38}O_{29}]^{5+}$ ($[M+5H]^{5+}$): 745.1219, found: 745.1224; calc. for $[C_{199}H_{338}N_{38}O_{29}]^{6+}$ ($[M+6H]^{6+}$): 621.1028, found: 621.1022.

H-^{h}Lys(NBD)-^{h}Lys$_2$-^{h}Trp$_2$-^{h}Val$_{19}$-^{h}Trp$_2$-^{h}Lys$_2$-NH$_2$ (10)

HPLC (MN Nucleodur® 100-5-C18, 250 mm × 21 mm, 5 µm, flow rate: 10 mL/min, gradient: 80-100% B in 40 min, λ in nm: 215, 464, 280): t_R = 30.00 min. **ESI-MS** (*m/z*): 769.3 $[M+5H]^{5+}$, 961.41 $[M+4H]^{4+}$, 1281.5 $[M+3H]^{3+}$. **HR-MS** (ESI): calc. for $[C_{203}H_{334}N_{41}O_{31}]^{3+}$ ($[M+3H]^{3+}$): 1281.5288, found: 1281.5290; calc. for $[C_{203}H_{335}N_{41}O_{31}]^{4+}$ ($[M+4H]^{4+}$): 961.3984, found: 961.3987; calc. for $[C_{203}H_{336}N_{41}O_{31}]^{5+}$ ($[M+5H]^{5+}$): 769.3202, found: 769.3202.

H-^{h}Lys$_2$-^{h}Trp$_2$-^{h}Val$_{19}$-^{h}Trp$_2$-^{h}Lys$_2$-^{h}Lys(TAMRA)-NH$_2$ (11)

HPLC (MN Nucleodur® 100-5-C18, 250 mm × 21 mm, 5 µm, flow rate: 10 mL/min, gradient: 90-100% B in 30 min, λ in nm: 215, 540, 280): t_R = 11.20 min. **ESI-MS** (*m/z*): 682.8 $[M+6H]^{6+}$, 819.1 $[M+5H]^{5+}$, 1023.7 $[M+4H]^{4+}$, 1364.6 $[M+3H]^{3+}$. **HR-MS** (ESI): calc. for $[C_{222}H_{353}N_{40}O_{32}]^{3+}$ ($[M+3H]^{3+}$): 1364.5756, found: 1364.5769; calc. for $[C_{222}H_{354}N_{40}O_{32}]^{4+}$ ($[M+4H]^{4+}$): 1023.6835, found: 1023.6845; calc. for $[C_{222}H_{355}N_{40}O_{32}]^{5+}$ ($[M+5H]^{5+}$): 819.1483, found: 819.1490; calc. for $[C_{222}H_{356}N_{40}O_{32}]^{6+}$ ($[M+6H]^{6+}$): 682.7915, found: 682.7915.

H-^{h}Lys(acetyl)-^{h}Lys$_2$-^{h}Trp$_2$-^{h}Val$_9$-^{h}Gln-^{h}Val$_5$-^{h}Gln-^{h}Val$_3$-^{h}Trp$_2$-^{h}Lys$_2$-NH$_2$ (12)

HPLC (MN Nucleodur® 100-5-C18, 250 mm × 21 mm, 5 µm, flow rate: 10 mL/min, gradient: 90-100% B in 30 min, λ in nm: 215, 254, 280): t_R = 14.12 min. **ESI-MS** (*m/z*): 630.8 $[M+6H]^{6+}$, 756.7 $[M+5H]^{5+}$, 945.6 $[M+4H]^{4+}$, 1260.5 $[M+3H]^{3+}$. **HR-MS** (ESI): calc. for $[C_{199}H_{333}N_{40}O_{31}]^{3+}$ ($[M+3H]^{3+}$): 1260.5251, found: 1260.5266; calc. for $[C_{199}H_{334}N_{40}O_{31}]^{4+}$ ($[M+4H]^{4+}$): 945.6457, found: 945.6457; calc. for $[C_{199}H_{335}N_{40}O_{31}]^{5+}$ ($[M+5H]^{5+}$): 756.7180, found: 756.7180; calc. for $[C_{199}H_{336}N_{40}O_{31}]^{6+}$ ($[M+6H]^{6+}$): 630.7662, found: 630.7664.

H-^{h}Lys(NBD)-^{h}Lys$_2$-^{h}Trp$_2$-^{h}Val$_9$-^{h}Gln-^{h}Val$_5$-^{h}Gln-^{h}Val$_3$-^{h}Trp$_2$-^{h}Lys$_2$-NH$_2$ (13)

HPLC (MN Nucleodur® 100-5-C18, 250 mm × 21 mm, 5 µm, flow rate: 10 mL/min, gradient: 85-100% B in 30 min, λ in nm: 215, 464, 280): t_R = 23.66 min. **ESI-MS** (*m/z*): 650.9 $[M+6H]^{6+}$, 780.9 $[M+5H]^{5+}$, 975.9 $[M+4H]^{4+}$, 1300.9 $[M+3H]^{3+}$. **HR-MS** (ESI): calc. for $[C_{203}H_{332}N_{43}O_{33}]^{3+}$ ($[M+3H]^{3+}$): 1300.8555, found: 1300.8583; calc. for $[C_{203}H_{333}N_{43}O_{33}]^{4+}$ ($[M+4H]^{4+}$): 975.8935, found: 975.8952;

calc. for $[C_{203}H_{334}N_{43}O_{33}]^{5+}$ ($[M+5H]^{5+}$): 780.9162, found: 780.9182; calc. for $[C_{203}H_{335}N_{43}O_{33}]^{6+}$ ($[M+6H]^{6+}$): 650.9314, found: 650.9315.

H-^{h}Lys$_2$-^{h}Trp$_2$-^{h}Val$_3$-^{h}Gln-^{h}Val$_5$-^{h}Gln-^{h}Val$_9$-^{h}Trp$_2$-^{h}Lys$_2$-^{h}Lys(TAMRA)-NH$_2$ (14)

HPLC (MN Nucleodur® 100-5-C18, 250 mm × 21 mm, 5 µm, flow rate: 10 mL/min, gradient: 86-100% B in 30 min, λ in nm: 215, 540, 280): t_R = 17.80 min. **ESI-MS** (*m*/*z*): 692.5 $[M+6H]^{6+}$, 830.7 $[M+5H]^{5+}$, 1038.2 $[M+4H]^{4+}$, 1383.9 $[M+3H]^{3+}$. **HR-MS** (ESI): calc. for $[C_{222}H_{351}N_{42}O_{34}]^{3+}$ ($[M+3H]^{3+}$): 1383.9024, found: 1383.9027; calc. for $[C_{222}H_{352}N_{42}O_{34}]^{4+}$ ($[M+4H]^{4+}$): 1038.1786, found: 1038.1790; calc. for $[C_{222}H_{353}N_{42}O_{34}]^{5+}$ ($[M+5H]^{5+}$): 830.7443, found: 830.7447; calc. for $[C_{222}H_{354}N_{42}O_{34}]^{6+}$ ($[M+6H]^{6+}$): 692.4549, found: 692.4548.

H-^{h}Lys(acetyl)-^{h}Lys$_2$-^{h}Trp$_2$-^{h}Val$_9$-^{h}Gln-^{h}Val$_2$-^{h}Gln-^{h}Val$_2$-^{h}Gln-^{h}Val$_3$-^{h}Trp$_2$-^{h}Lys$_2$-NH$_2$ (15)

HPLC (MN Nucleodur® 100-5-C18, 250 mm × 21 mm, 5 µm, flow rate: 10 mL/min, gradient: 90-100% B in 30 min, λ in nm: 215, 254, 280): t_R = 15.22 min. **ESI-MS** (*m*/*z*): 635.6 $[M+6H]^{6+}$, 762.5 $[M+5H]^{5+}$, 952.9 $[M+4H]^{4+}$, 1270.2 $[M+3H]^{3+}$. **HR-MS** (ESI): calc. for $[C_{199}H_{332}N_{41}O_{32}]^{3+}$ ($[M+3H]^{3+}$): 1270.1885, found: 1270.1913; calc. for $[C_{199}H_{333}N_{41}O_{32}]^{4+}$ ($[M+4H]^{4+}$): 952.8932, found: 952.8952; calc. for $[C_{199}H_{334}N_{41}O_{32}]^{5+}$ ($[M+5H]^{5+}$): 762.5160, found: 762.5183; calc. for $[C_{199}H_{335}N_{41}O_{32}]^{6+}$ ($[M+6H]^{6+}$): 635.5979, found: 635.5982.

H-^{h}Lys(NBD)-^{h}Lys$_2$-^{h}Trp$_2$-^{h}Val$_9$-^{h}Gln-^{h}Val$_2$-^{h}Gln-^{h}Val$_2$-^{h}Gln-^{h}Val$_3$-^{h}Trp$_2$-^{h}Lys$_2$-NH$_2$ (16)

HPLC (MN Nucleodur® 100-5-C18, 250 mm × 21 mm, 5 µm, flow rate: 10 mL/min, gradient: 85-100% B in 30 min, λ in nm: 215, 464, 280): t_R = 23.38 min. **ESI-MS** (*m*/*z*): 655.8 $[M+6H]^{6+}$, 786.7 $[M+5H]^{5+}$, 983.1 $[M+4H]^{4+}$, 1310.5 $[M+3H]^{3+}$. **HR-MS** (ESI): calc. for $[C_{203}H_{331}N_{44}O_{34}]^{3+}$ ($[M+3H]^{3+}$): 1310.5189, found: 1310.5194; calc. for $[C_{203}H_{332}N_{44}O_{34}]^{4+}$ ($[M+4H]^{4+}$): 983.1410, found: 983.1415; calc. for $[C_{203}H_{333}N_{44}O_{34}]^{5+}$ ($[M+5H]^{5+}$): 786.7143, found: 786.7150; calc. for $[C_{203}H_{334}N_{44}O_{34}]^{6+}$ ($[M+6H]^{6+}$): 655.7631, found: 655.7627.

H-^{h}Lys$_2$-^{h}Trp$_2$-^{h}Val$_3$-^{h}Gln-^{h}Val$_2$-^{h}Gln-^{h}Val$_2$-^{h}Gln-^{h}Val$_9$-^{h}Trp$_2$-^{h}Lys$_2$-^{h}Lys(TAMRA)-NH$_2$ (17)

HPLC (MN Nucleodur® 100-5-C18, 250 mm × 21 mm, 5 µm, flow rate: 10 mL/min, gradient: 85-100% B in 30 min, λ in nm: 215, 540, 280): t_R = 18.57 min. **ESI-MS** (*m*/*z*): 697.3 $[M+6H]^{6+}$, 836.5 $[M+5H]^{5+}$, 1045.4 $[M+4H]^{4+}$, 1393.6 $[M+3H]^{3+}$. **HR-MS** (ESI): calc. for $[C_{222}H_{350}N_{43}O_{35}]^{3+}$ ($[M+3H]^{3+}$): 1393.5658, found: 1393.5667; calc. for $[C_{222}H_{351}N_{43}O_{35}]^{4+}$ ($[M+4H]^{4+}$): 1045.4261, found:

1045.4271; calc. for $[C_{222}H_{352}N_{43}O_{35}]^{5+}$ ($[M+5H]^{5+}$): 836.5424, found: 836.5435; calc. for $[C_{222}H_{353}N_{43}O_{35}]^{6+}$ ($[M+6H]^{6+}$): 697.2865, found: 697.2874.

CD-spectra of the β-peptides 9, 12 and 15 in TFE

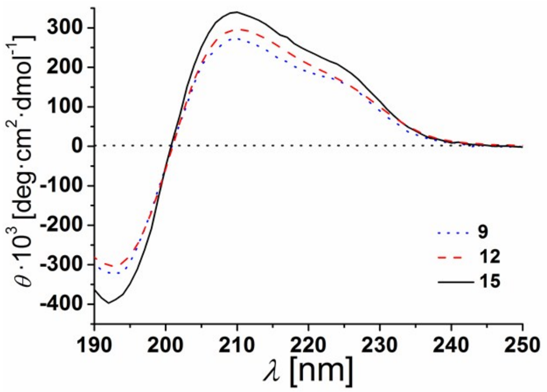

Figure S2: CD-spectra of **9**, **12** and **15** in TFE (peptides concentration: 38 μM, 25 °C).

CD-spectra of the β-peptides 12 and 15 in at the P/L-ratio = 1/500 at 25 °C and 60 °C

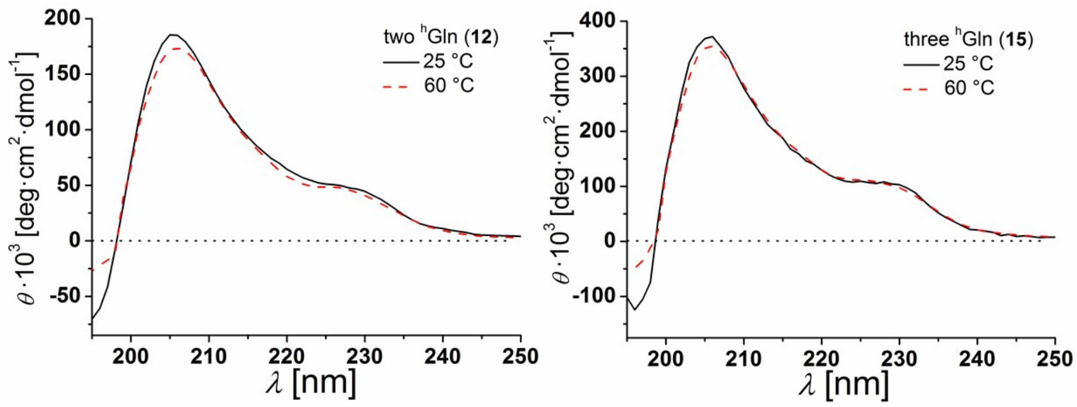

Figure S3: CD-spectra of **12** (left) and **15** (right) in DOPC LUVs (peptides concentration: 38 μM, P/L-ratio = 1/500, 25 °C and 60 °C).

Concentration dependent fluorescence spectra of the β-peptides 9-11, 12-14 and 15-17 at the P/L-ratio = 1/500 at 25 °C and 60 °C

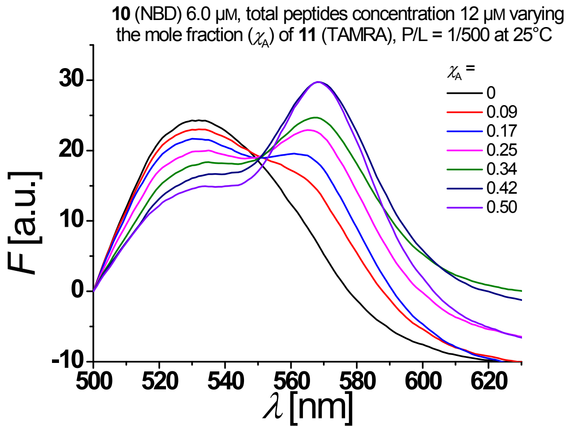

Figure S4: Concentration dependent fluorescence spectra of compound **10** (NBD) at 6.0 µM with varying amounts of compound **11** (TAMRA) in DOPC LUVs at 25 °C. The P/L-ratio of 1/500 and the total peptide concentration of 12 µM were kept constant by addition of the non-labelled peptide **9**. The data points at 530 nm were used in the normalized fluorescence emission plots as a function of χ_A (Fig. 6C).

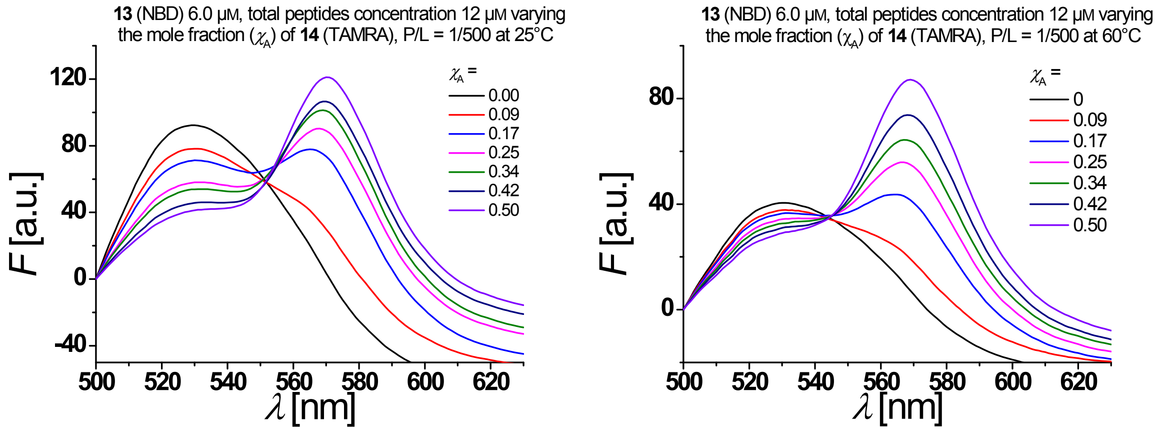

Figure S5: Concentration dependent fluorescence spectra of compound **13** (NBD) at 6.0 µM with varying amounts of compound **14** (TAMRA) in DOPC LUVs at 25 °C (left) and 60 °C (right). The P/L-ratio of 1/500 and the total peptide concentration of 12 µM were kept constant by addition of the non-labelled peptide **12**. The data points at 530 nm (25 °C and 60 °C) were used in the normalized fluorescence emission plots as a function of χ_A (Fig. 6C, Fig. 7, Fig. S9).

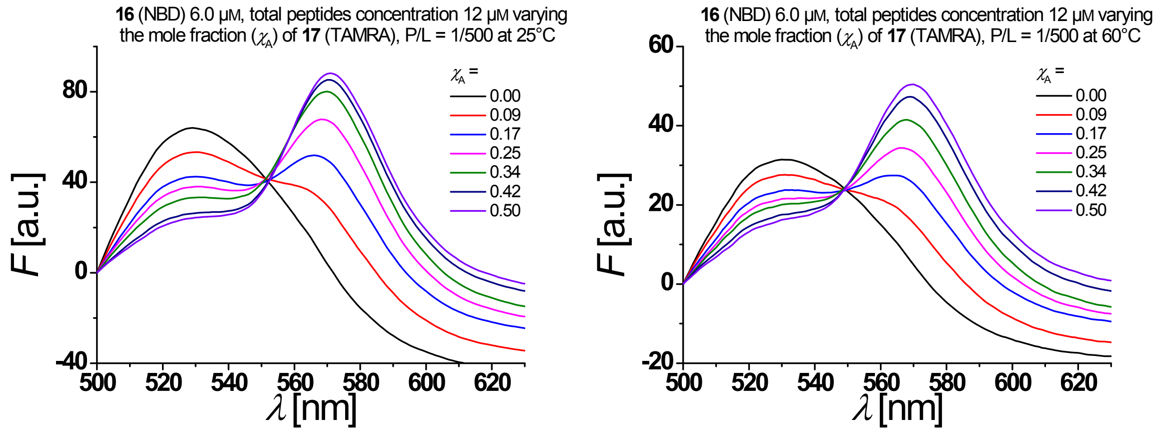

Figure S6: Concentration dependent fluorescence spectra of compound **16** (NBD) at 6.0 μM with varying amounts of compound **17** (TAMRA) in DOPC LUVs at 25 °C (left) and 60 °C (right). The P/L-ratio of 1/500 and the total peptide concentration of 12 μM were kept constant by addition of the non-labelled peptide **15**. The data points at 530 nm (25 °C and 60 °C) were used in the normalized fluorescence emission plots as a function of χ_A (Fig. 6C, Fig. 7, Fig. S9).

Concentration dependent fluorescence spectra of the β-peptides 12-14 and 15-17 at the P/L-ratios = 1/750 and 1/1000 at 25 °C

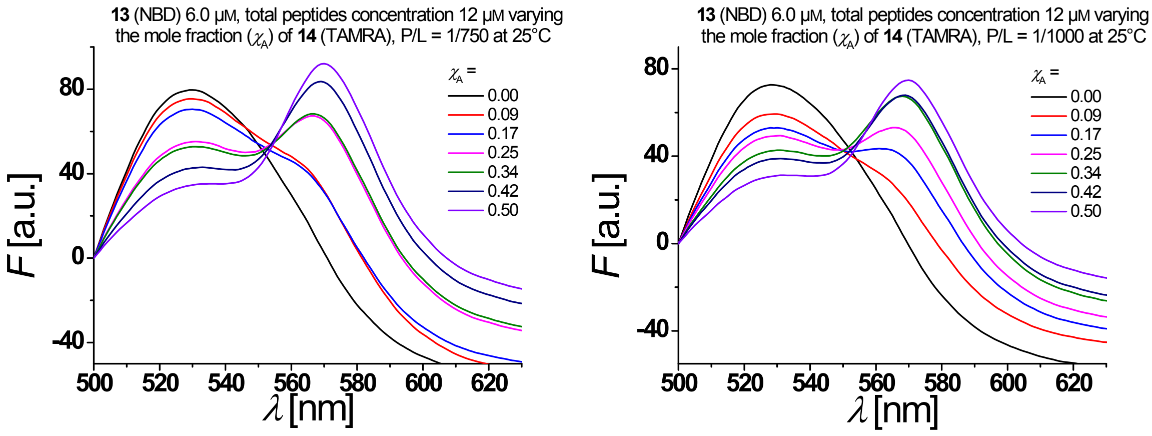

Figure S7: Concentration dependent fluorescence spectra of compound **13** (NBD) at 6.0 µM with varying amounts of compound **14** (TAMRA) in DOPC LUVs at 25 °C. The P/L-ratio of 1/750 (left) or 1/1000 (right) and the total peptide concentration of 12 µM were kept constant by addition of the non-labelled peptide **12**. The data points at 530 nm were used in the normalized fluorescence emission plots as a function of χ_A (Fig. S9).

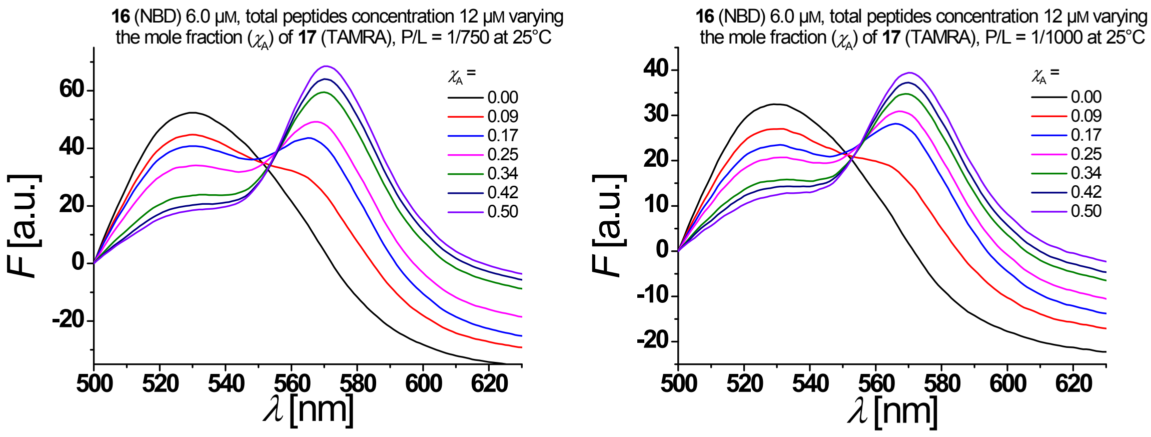

Figure S8: Concentration dependent fluorescence spectra of compound **16** (NBD) at 6.0 µM with varying amounts of compound **17** (TAMRA) in DOPC LUVs at 25 °C. The P/L-ratio of 1/750 (left) or 1/1000 (right) and the total peptide concentration of 12 µM were kept constant by addition of the non-labelled peptide **15**. The data points at 530 nm were used in the normalized fluorescence emission plots as a function of χ_A (Fig. S9).

Relative changes in donor-fluorescence emission (F/F_0) as a function of increasing acceptor concentration (χ_A)

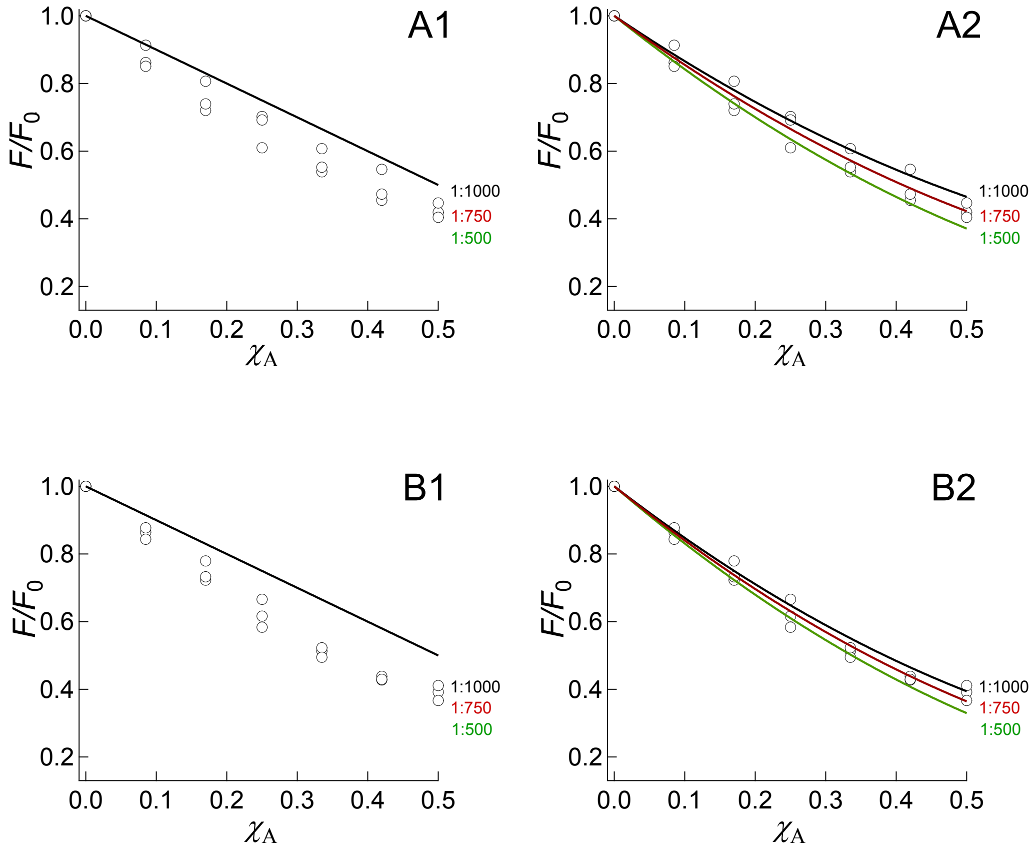

Figure S7. FRET analysis of **A.** two ^{h}Gln and **B.** three ^{h}Gln in 100 nm DOPC vesicles. The relative changes in NBD-fluorescence emission (F/F_0) as a function of increasing acceptor concentration χ_A are plotted. The solid lines are the results of a global fit analysis assuming (1) a monomer-dimer and (2) a monomer-trimer equilibrium. The FÖRSTER radius R_0 was determined to be R_0 = 5.1 nm as obtained from results with the β-peptides without recognition units assuming that only monomers are in the membrane and the FRET occurs statistically.[15,16] A monomer-dimer equilibrium does not explain the data. Even the assumption of a pure dimer (solid black line) does not explain the observed plots. For a monomer-trimer equilibrium, the following dissociation constants were obtained for two ^{h}Gln: A2: K_D = (17.2 ± 7.0)·10^{-8} MF^2 and three ^{h}Gln: B2: K_D = (4.4 ± 4.3)·10^{-8} MF^2.

1 B. Bilgiçer and K. Kumar, *Proc. Natl. Acad. Sci. U. S. A.*, 2004, **101**, 15324–15329.
2 A. Pingoud and C. Urbanke, *Arbeitsmethoden der Biochemie*, Walter de Gruyter, 1997.
3 N. J. Greenfield, *Nat. Protoc.*, 2007, **1**, 2876–2890.
4 G. Guichard, S. Abele and D. Seebach, *Helv. Chim. Acta*, 1998, **81**, 187–206.
5 P. I. Arvidsson, J. Frackenpohl and D. Seebach, *Helv. Chim. Acta*, 2003, **86**, 1522–1553.
6 B. S. Patil, G.-R. Vasanthakumar and V. V. S. Babu, *Lett. Pept. Sci.*, 2002, **9**, 231–233.
7 Andrew G. Myers and James L. Gleason, *Org. Synth.*, 1999, **76**, 57–76.
8 P. Marfey, *Carlsberg Res. Commun.*, 1984, **49**, 591–596.
9 R. Bhushan and H. Brückner, *Amino Acids*, 2004, **27**, 231–247.
10 R. Bhushan, H. Brückner and V. Kumar, *Biomed. Chromatogr.*, 2007, **21**, 1064–1068.
11 *Novabiochem® Peptide Synthesis*, Peptide synthesis protocols, 2012.
12 M. Gude, J. Ryf and P. D. White, *Lett. Pept. Sci.*, 2002, **9**, 203–206.
13 B. Blankemeyer-Menge, M. Nimtz and R. Frank, *Tetrahedron Lett.*, 1990, **31**, 1701–1704.
14 H. Wu, Y. Li, G. Bai, Y. Niu, Q. Qiao, J. D. Tipton, C. Cao and J. Cai, *Chem. Commun.*, 2014, **50**, 5206–5208.
15 P. E. Schneggenburger, S. Müllar, B. Worbs, C. Steinem and U. Diederichsen, *J. Am. Chem. Soc.*, 2010, **132**, 8020–8028.
16 P. K. Wolber and B. S. Hudson, *Biophys. J.*, 1979, **28**, 197–210.
